# Elucidation of the anti-hyperammonemic mechanism of *Lactobacillus amylovorus* JBD401 by comparative genomic analysis

**DOI:** 10.1186/s12864-018-4672-3

**Published:** 2018-04-25

**Authors:** Parul Singh, Hea-Jong Chung, In-Ah Lee, Roshan D’Souza, Hyeon-Jin Kim, Seong-Tshool Hong

**Affiliations:** 1 Department of Biomedical Sciences and Institute for Medical Science, Chonbuk National University Medical School, Jeonju, Chonbuk 54907 South Korea; 2 JINIS BDRD institute, JINIS Biopharmaceuticals Co., 913 Gwahak-Ro, Bongdong, Wanju, Chonbuk 55321 South Korea; 3 Present address: Department of Chemistry, Gunsan National University, Gunsan, Chonbuk 51450 South Korea

**Keywords:** Hyperammonemia, *Lactobacillus amylovorus* JBD401, Comparative genome analysis, Ammonia assimilation

## Abstract

**Background:**

Recent experimental evidence showed that lactobacilli could be used as potential therapeutic agents for hyperammonemia. However, lack of understanding on how lactobacilli reduce blood ammonia levels limits application of lactobacilli to treat hyperammonemia.

**Results:**

We report the finished and annotated genome sequence of *L. amylovorus* JBD401 (GenBank accession no. CP012389). *L. amylovorus* JBD401 reducing blood ammonia levels dramatically was identified by high-throughput screening of several thousand probiotic strains both within and across *Lactobacillus* species *in vitro*. Administration of *L. amylovorus* JBD401 to hyperammonemia-induced mice reduced the blood ammonia levels of the mice to the normal range. Genome sequencing showed that *L. amylovorus* JBD401 had a circular chromosome of 1,946,267 bp with an average GC content of 38.13%. Comparative analysis of the *L. amylovorus* JBD401 genome with *L. acidophilus* and *L. amylovorus* strains showed that *L. amylovorus* JBD401 possessed genes for ammonia assimilation into various amino acids and polyamines Interestingly, the genome of *L. amylovorus* JBD401 contained unusually large number of various pseudogenes suggesting an active stage of evolution.

**Conclusions:**

*L. amylovorus* JBD401 has genes for assimilation of free ammonia into various amino acids and polyamines which results in removal of free ammonia in intestinal lumen to reduce the blood ammonia levels in the host. This work explains the mechanism of how probiotics reduce blood ammonia levels.

**Electronic supplementary material:**

The online version of this article (10.1186/s12864-018-4672-3) contains supplementary material, which is available to authorized users.

## Background

Ammonia is produced as a byproduct from the metabolism of nitrogenous compounds such as amino acids, purine/pyrimidine bases, and amines in mammals. Because ammonia is a strong neurotoxin, it is converted to urea in the liver prior to excretion in urine [1]. Hyperammonemia is induced when the blood concentration of ammonia is elevated as the result of a disturbance in ammonia metabolism. The induction of hyperammonemia can be congenital, resulting from a genetic defect in the enzymes in the urea cycle, or acquired, resulting from liver failure involving the urea cycle [2]. The neurotoxic nature of ammonia leads hyperammonemia to result in encephalopathy, which is characterized by vomiting, hypotonia, lethargy, seizures, and coma [3]. These serious clinical consequences of hyperammonemia have motivated various approaches to reduce the blood concentration of ammonia by limited protein intake, administration of lactulose or neomycin, hemodialysis, plasmapheresis, and gut cleansing [4]. However, these approaches are not very effective in the treatment of hyperammonemia and can be used only in an emergency situation because of the complexity of the treatments.

Previous research on association between ammonia levels and Alzheimer’s disease (AD) had found that the blood ammonia levels in AD patients were significantly higher than in control groups [5, 6], indicating that the neurotoxic nature of ammonia contribute to the etiology of AD. The possible etiological association of ammonia to AD was further solidated by finding that Mediterrian diet which consists of an unusally large quantity of lactobacilli prevented AD significantly [7, 8]. Considering the neurotoxic nature of ammonia, a positive correlation between blood ammonia levels and AD is not a surprising phenomenon.

The intestine is the site where digested nutrients are absorbed through intestinal capillaries and lymphatic vessels [9]. However, the metabolites do not always flow in one direction, i.e.*,* from the intestinal lumen to intestinal capillaries or lymphatic vessels [10]. Ammonia is an example of a metabolite that does not move in a single direction; the intestine secretes and absorbs ammonia through both passive diffusion and specific transporters such as RhBG (Rh family, B glycoprotein) and RhCG (Rh family, C glycoprotein) [11]. Although not widely recognized, the intestine is a major site of ammonia transport [12]. Because ammonia can move bidirectionally between the intestinal lumen and body fluid, removal of ammonia in the intestinal lumen by intestinal microbes such as *Lactobacillus* species could reduce the blood concentration of ammonia, thus providing an ideal treatment method for hyperammonemia.

Although it is a promising approach, administration of lactobacilli to treat hyperammonemia [13] has not been adopted in medical practice yet. There are two problems in adopting a *Lactobacillus* strain as a therapeutic agent to treat hyperammonemia. The action mechanism on how lactobacilli reduce blood ammonia levels has to be established and an ideal strain for hyperammonemia needs to be identified. In this work, we screened several thousand probiotic strains both within and across *Lactobacillus* species and identified *L. amylovorus* JBD401 as a strain that most efficiently removed ammonia from the surrounding medium. Administration of *L. amylovorus* JBD401 into the hyperammonemia-induced mice reduced the blood ammonia levels to the normal range. Genome sequencing showed that *L. amylovorus* JBD401 has a complete set of genes required for ammonia assimilation into various amino acids and polyamines. Possession of the pathways for ammonia assimilation should enable *L. amylovorus* JBD401 to remove ammonia from the intestinal lumen, thereby eventually reducing the blood concentration of ammonia. This work not only identifies the best candidate strain for treatment of hyperammonemia, but also sheds light on the unsolved mechanism by which *Lactobacillus* reduce blood ammonia levels.

## Results

### Identification of a *Lactobacillus* strain, *L. amylovorus* JBD401, for treatment of hyperammonemia

**Fig. 1:**
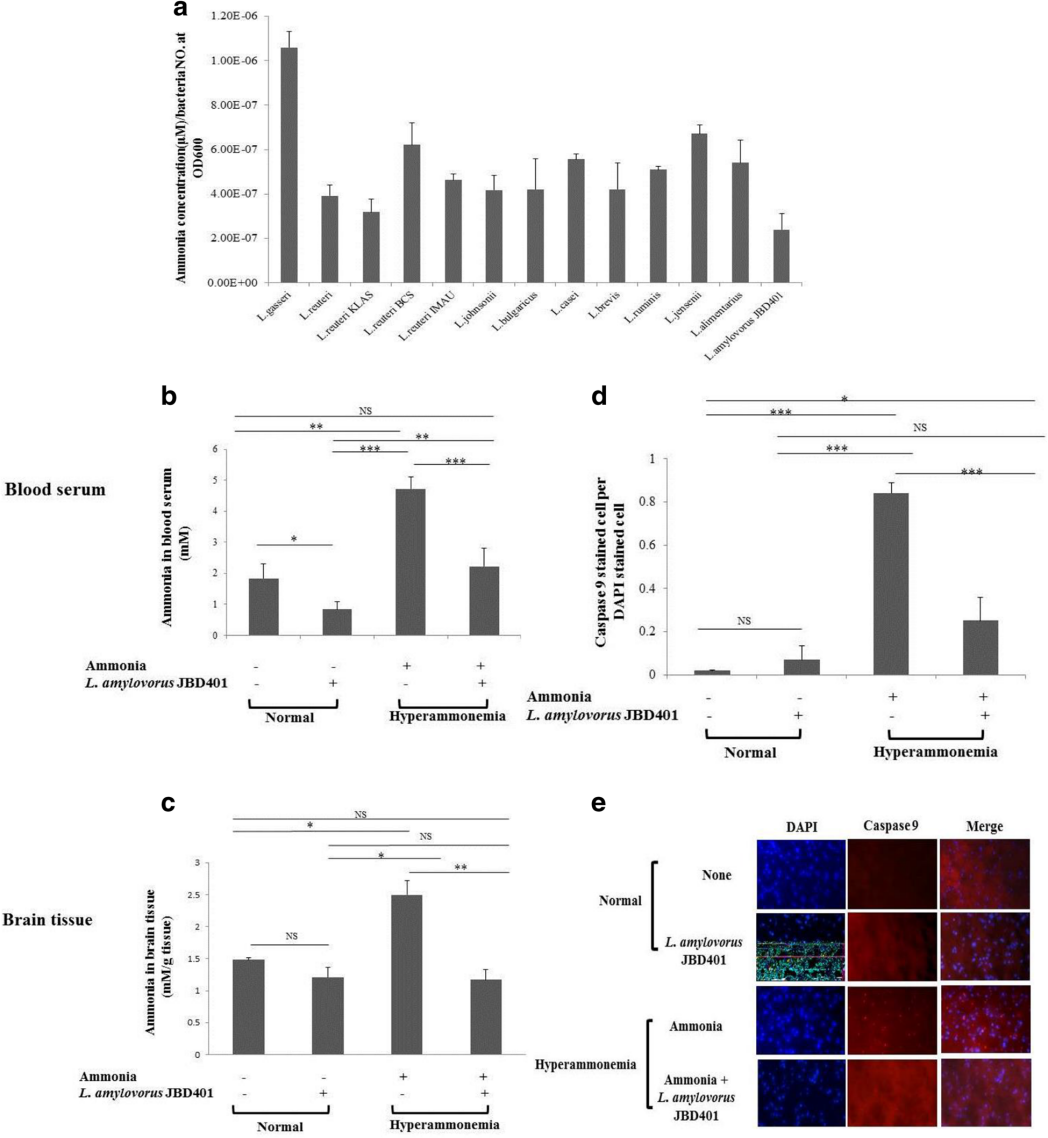
*Lactobacillus amylovorus* JBD401 reduced blood ammonia levels efficiently. **a** In vitro high throughput screening results showing promising probiotic strains for hyperammonemia. *L. amylovorus* JBD401 was especially worth noting as a candidate strain to treat hyperammonemia **b**, **c** After hyperammonemia was induced in the mouse-fed *L. amylovorus* JBD401, ammonia levels in its blood and brain tissue were analyzed. **d**, **e** After hyperammonemia was induced in the mouse-fed *L. amylovorus* JBD401, caspase 9 activity in brain tissue were analyzed. All data were expressed as the mean ± SD*,* as indicated. The statistical comparisons were analyzed using an unpaired Student’s t-test*.* All differences were considered statistically significant if *p <* 0.05. Statistical significance is shown as *p < 0.05, **p < 0.01 and ***p < 0.001, ^NS^*p* > 0.05

Recent findings revealed a possibility that oral administration of certain *Lactobacillus* strains to hyperammonemic patients could reduce the ammonia concentration in blood [14, 15]. On the basis of these findings we extensively screened probiotic strains from the gutmicrobiotabank including *Lactobacillus*, *Lactococcus* and *Streptococcus* (https://www.gutmicrobiotabank.com)*.* By high-throughput screening of several thousand probiotic strains *in vitro*, we identified several probiotic strains that efficiently removed ammonia from their surrounding culture medium, suggesting a possibility of development of the probiotic strains as therapeutic agents for treatment of hyperammonemia (Fig. 1a). Especially, *L. amylovorus* JBD401 was notable in terms of removing ammonia from its surrounding culture medium. In vivo experiments showed that *L. amylovorus* JBD401 significantly reduced the ammonia concentrations in the blood of hyperammonemic mice (Fig. 1b). The blood ammonia concentrations were decreased to half of the original values in hyperammonemia-induced mice fed *L. amylovorus* JBD401 for 7 days, whereas untreated hyperammonemic mice maintained abnormally high blood ammonia concentrations. In addition to reduction of blood ammonia levels, administration of *L. amylovorus* JBD401 decreased the ammonia concentrations in the brains of the hyperammonemic mice to the same extent as blood ammonia levels (Fig. 1c). These animal experiments clearly showed that *L. amylovorus* JBD401 could be developed as an ideal therapeutic agent for treatment of hyperammonemia.

It is known that ammonia causes neuronal apoptosis by activating the caspase 9-dependent apoptotic pathway [16]. To confirm whether removal of ammonia in the brain by *L. amylovorus* JBD401 actually inhibits ammonia-induced neurotoxicity, mouse brain tissues were analyzed using the Fluorescent-Labeled Inhibitor of Caspases (FLICA™) assay to detect the induction levels of caspases. As shown in Fig. 1d and Fig. 1e, the caspase 9-dependent apoptotic pathway was activated in the control hyperammonemic mice, but was not activated in the hyperammonemic mice fed *L. amylovorus* JBD401. Therefore, it is reasonable to conclude that *L. amylovorus* JBD401 assimilated ammonia in the mouse intestine to reduce blood ammonia levels in the hyperammonemic mice, and that feeding with *L. amylovorus* JBD401 protects neurons against the ammonia-induced neurotoxicity.

### Genome features of *L. amylovorus* JBD401

**Fig. 2:**
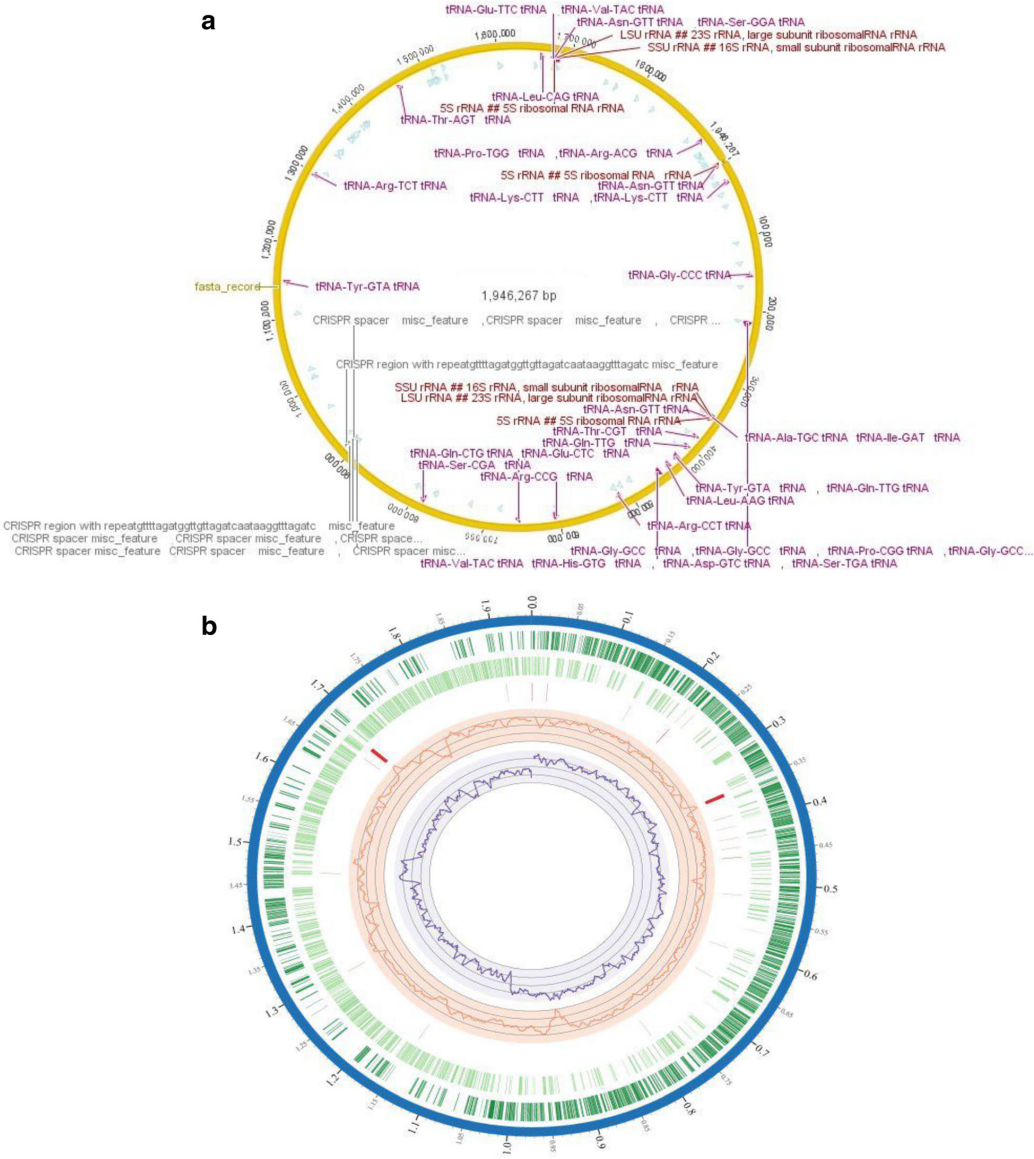
Circular view of the complete genome sequence of *Lactobacillus amylovorus* JBD401. **a** The circle was created by using Geneious 7.0.6. Genome of *L. amylovorus* JBD401 revealed total 35 tRNAs and 7 rRNAs. Some tRNAs were redundant. Regardless of that, they represented 16 amino acid biosynthetic pathways except methionine, phenylalanine, tryptophan and cysteine. **b** Genome atlas of *L. amylovorus* JBD401.This atlas represents a circular view of the complete genome and was created using PATRIC. List of rings, from outside to inside 1) Chromosomes, plasmids, contigs, 2) CDS forward strand, 3) CDS reverse strand, 4) RNAs 5) GC Content line Plot and 6) GC Skew line Plot

After identifying *L. amylovorus* JBD401 as a potential strain for treatment of hyperammonemia, we sequenced the whole genome of *L. amylovorus* JBD401 to elucidate how this *Lactobacillus* strain reduces blood ammonia levels. The genome sequence of *L. amylovorus* JBD401 revealed a circular chromosome of 1,946,267 bp with an average GC content of 38.13%. The complete genome sequence was deposited in the GenBank database (Accession no. CP012389). The genome contained 2244 ORFs predicted by GLIMMER, but manual annotation resulted in 1979 protein encoding genes, representing a coding density of 84.17%. Seven rRNAs (consisting of 5 s, 16S, and 23S), one non-coding RNA, and 35 tRNAs were distributed throughout the genome despite its modest size (Fig. 2).

Whole-genome sequencing of *L. amylovorus* JBD401 confirmed the presence of the complete pathway of purine metabolism with all required genes (GAR, FGAR, FGAM, AIR, CAIR, SAICAR, AICAR, FAICR, and IMP), whereas only a partial pathway was available for pyrimidine metabolism. Two genes among 10 genes required for de novo pyrimidine metabolism were lacking. Interestingly, *L. amylovorus* JBD401 appears to assimilate ammonia through multiple pathways other than the well-known pathway of synthesizing Gln from Glu via glutamine synthetase I (glnA, AB283_1841). Gln synthesis seems to also be catalyzed by amidophosphoribosyltransferase (AB283_1899) upon entering the purine metabolism pathway (Additional file 1: see supplementary results “Genome features of *Lactobacillus amylovorus* JBD401*”*). *L. amylovorus* JBD401 possesses L-asparaginase (AB283_2020) that can synthesize Asp from Asn. The presence of asparagine synthetase (AB283_0122) in *L. amylovorus* JBD401 suggests that interconversion between Gln (Glutamine) and Glu (Glutamic Acid) is possible. In addition, *L. amylovorus* JBD401 seems to be capable of de novo synthesis of six amino acids: Asp (Aspartic Acid), Gln, Gly (Glycine), Ser (Serine), Thr (Threonine), and Cys (Cysteine). *L. amylovorus* JBD401 seems to be able to synthesize most, but not all, cofactors and vitamins. Overall, *L. amylovorus* JBD401 has certain degree of auxotrophy for nucleotides, amino acids, cofactors, and vitamins.

**Fig. 3:**
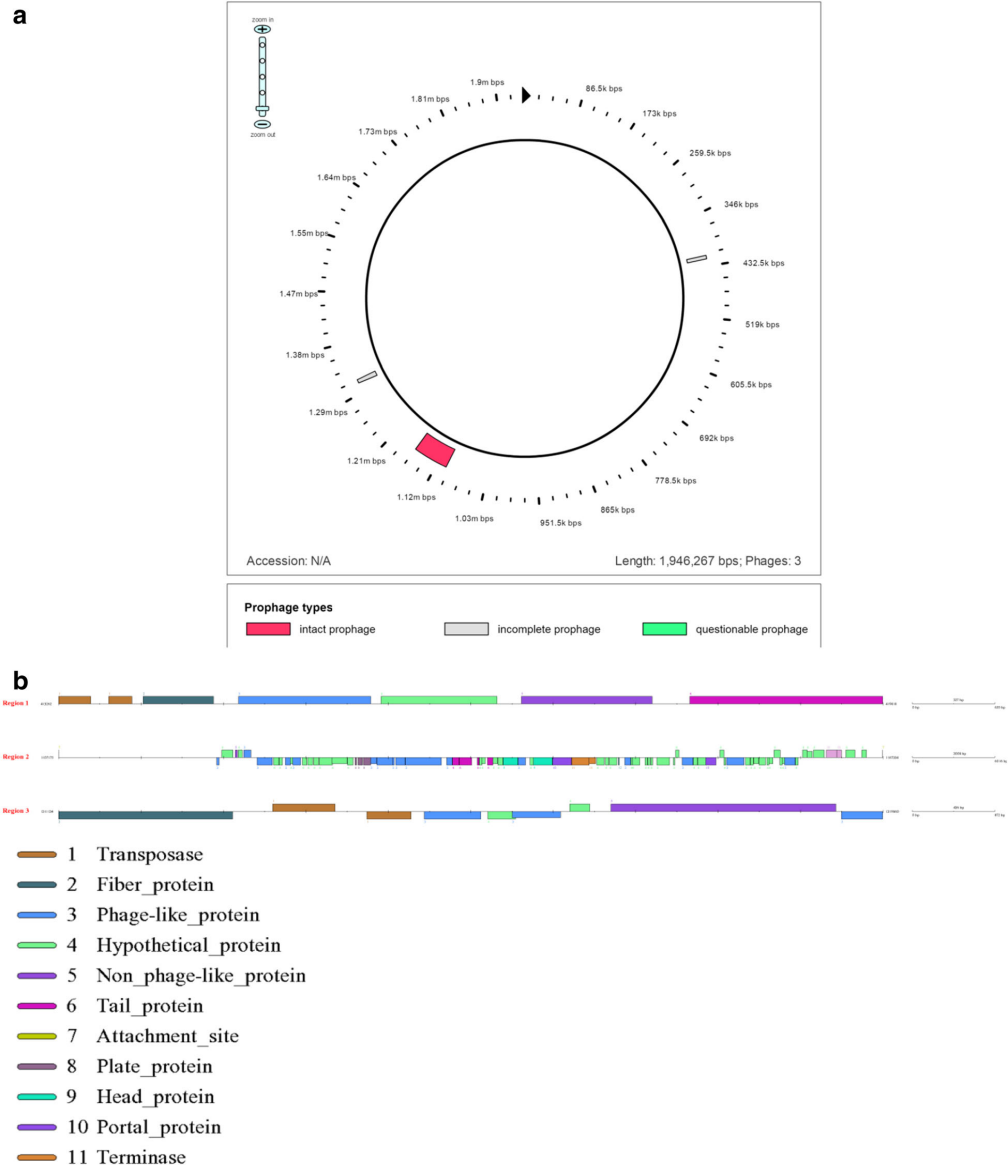
Prophages in the *Lactobacillus amylovorus* JBD401 genome. **a** Circular view of prophages in the *L. amylovorus* JBD401 genome; incomplete prophages shown in grey color and complete prophage are in pink color. One incomplete prophage resides in 346 kbps to 432.5 kbps and another incomplete prophage found in 1.29 mbps to 1.38 mbps. The complete prophage spans residues 1.03 mbps to 1.21 mbps. **b** The prophage regions in the *L. amylovorus* JBD401 genome was divided into three regions and each region has several prophage remnants. Region 1 (6.5 kb, 413,262 bp–419,818 bp) and Region 3 (8.7 kb, 1,311,134 bp–1,319,860 bp) are smaller. The prophage had 7 and 9 cds with 40.20% and 41.55% GC content respectively. Region 2 (60.1 kb, 1,107,175 bp–1,167,322 bp) is longest among all the regions with 78 CDS with 35.16% GC content

Three prophages were found in the *L. amylovorus* JBD401 genome, of which two were incomplete and one was intact (Fig. 3). The intact prophage showed similarity to dairy *Lactobacillus helveticus* bacteriophage phiAQ113 [17]. The genome of phiAQ113 phage is related to the genomes of phage KC5a (present in *L. gasseri*) and phage Lj771 (present in *L. johnsonii*) [18]. These phylogenetic similarities suggest a probable common ancestral origin. As expected, Virulence finder [19] did not show any virulence factors in *L. amylovorus* JBD401, indicating that *L. amylovorus* JBD401 is safe from outbreak of verocytotoxin-producing *Escherichia coli* (VTEC) infections. CRISPR genes provide immunity to the host against phage, plasmid proliferation and genetic transformation. These genes are widely found in bacteria; to date, almost 40% of bacterial genomes have been reported to contain CRISPR genes. Two CRISPR genes (Csn1 family) were identified at location 907,737–908,783 bp, and two other CRISPR genes (cas1 and cas2) were present at 912098–913006 bp and 913,014–913,289 bp, respectively. We recognized six bacteriocins in *L. amylovorus* JBD401, among which three were clustered together at AB283_27–AB283_29, and three were scattered throughout the genome at AB283_0383, AB283_1910, and AB283_2114. Among the six genes for bacteriocins, five are involved in production and processing of helveticin J, while one encodes ABC-transporter auxiliary bacteriocin (Additional file 1: see supplementary results “Genome features of *Lactobacillus amylovorus* JBD401*”*). We found that genome of *L. amylovorus* JBD401 has total 16 protein sequences that are antibiotic resistant. Among them one sequence has similarity above cutoff values (97.65%) which is resistant to tetracycline and other 15 sequences have similarity below cutoff (Additional file 1: Table S7). We also found that carbamate kinase (CK) (EC 2.7.2.2) and ornithine carbamoyltransferase (OTC) (EC 2.1.3.3) were resistant to cephalosporin_i, penicillin and methicillin (Additional file 1: Table S8).

Generally, LAB (Lactic acid bacteria) has a limited ability to synthesize amino acids, and our genome analysis of *L. amylovorus* JBD401 showed that *L. amylovorus* JBD401 is no exception. Although LAB is believed to have relatively weak proteolytic systems compared to other bacteria, they are still sufficiently complex and capable of hydrolyzing proteins from food. In silico analysis of *L. amylovorus* JBD401 revealed the presence of a total of 53 proteases/peptidases and their related transporter proteins. ABC type transporters (OppA, OppB, OppC, OppD, and OppF) were identified as transporters for amino acids or oligopeptides (Additional file 1: Table S1). The proteases/peptidases of *L. amylovorus* JBD401 were grouped according to function and proposed family (Additional file 1: Table S2). The most dominant type of protease/peptidase was M (metallo) with 14 members, followed by S (serine) with 12 members and C (cysteine) with 11 members. The group of type A (aspartic) had only 2 members, and type T (threonine) had just 1 member. LAB is known to be capable of using almost all carbohydrates such as mono/di/polysaccharides. Raffinose and fructooligosaccharides (FOS) are complex dietary carbohydrates that are not digested in the upper GI tract of humans [20]. The *L. amylovorus* JBD401 genome possessed nine phosphoenolpyruvate sugar-transferase systems (PTS) and some ABC transporters, with six different families for FOS, ribose, maltose, sugar, and raffinose as well as some uncharacterized carbohydrates (Additional file 1: Table S3; Table S4). Interestingly, *L. amylovorus* JBD401 possessed genes for only partial biosynthesis of nicotinate, nicotinamide, pantothenate, CoA, folate, and retinol.

### Comparative genomics and functional analysis of *L. amylovorus* JBD401

The JBD401 strain was originally classified as a strain of *L. acidophilus* based on its morphological and biochemical characteristics but functional annotation of JBD401 clearly indicates that the genome sequence may have originated from a strain of *L. amylovorus.* Subsequently, we performed average nucleotide identity (ANI) test [21] and the result suggested *L. amylovorus* rather than *L. acidophilus* for this genome. The sequence of JBD401 genome was 98.727% identical by ANI to the type genome of *L. amylovorus*, with 85.0% coverage of the genome. Therefore, JBD401 was reclassified as *L. amylovorus* JBD401. We compared the genome of *L. amylovorus* JBD401 with the genomes of known *L. acidophilus* strains and *L. amylovorus* strains to elucidate how *L. amylovorus* JBD401 reduces blood ammonia levels. Every *L. acidophilus* and *L. amylovorus* genome sequenced to date (i.e., *L. acidophilus* NCFM, *L. acidophilus* 30SC, *L. acidophilus* La-14, *L. acidophilus* FSI4, *L. amylovorus* 30SC, *L. amylovorus* GRL1118 and *L. amylovorus* DSM20531) [22–26] showed similar genome size and numbers of coding genes, rRNAs, and tRNAs at the level of summary statistics (Additional file 1: Table S5; Additional file 1: Table S6). Interestingly, *L. amylovorus* JBD401 had uniquely large number of pseudogenes unlike other *L. acidophilus* and *L. amylovorus* strains*. L. amylovorus* JBD401 had 372 pseudogenes constituting 15.82% of the genome possibly indicative of ongoing specialization [27]. There are three main ways for pseudogenes to form in bacteria: degradation preceded by duplication, disruption by transposons and failed/incomplete horizontal gene transfer (HGT). HGT is the most common cause of pseudogenization [28].

**Fig. 4:**
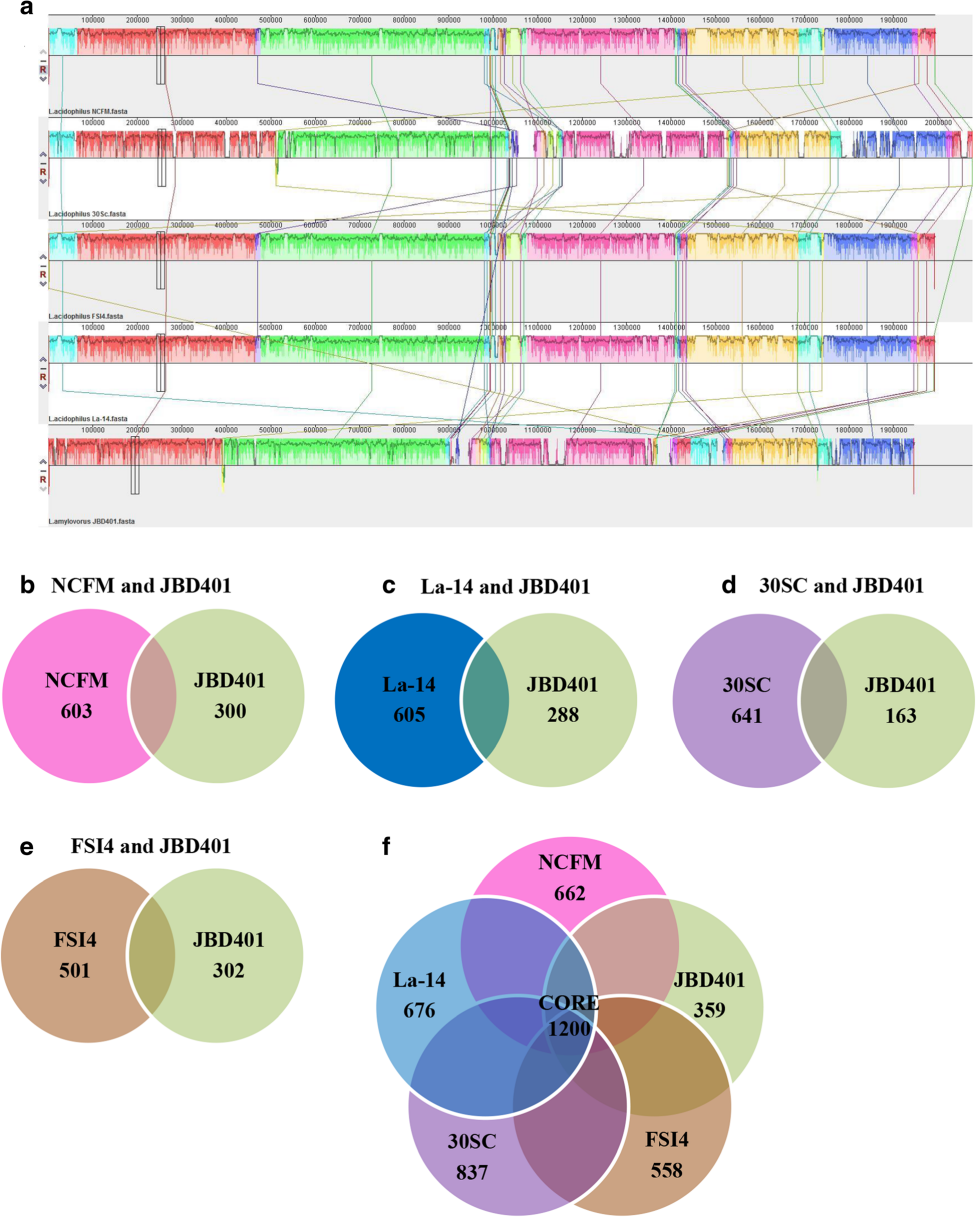
Comparative genome analysis of *Lactobacillus amylovorus* JBD401 with *Lactobacillus acidophilus* strains. **a** Multiple genome alignment of *L. acidophilus* NCFM, *L. acidophilus* 30SC, *L. acidophilus* FSI4, *L. acidophilus* La-14 and *L. amylovorus* JBD401 from top to bottom. Image was generated by Mauve. A scale is showing name of each genome sequence compared. The similar colored blocks of each genome are connected through lines representing homologous regions of each genome and non-homologous regions unique to each genome are white. **b** Core genes between *L. acidophilus* NCFM and *L. amylovorus* JBD401. **c** Core genes between *L. acidophilus* La-14 and *L. amylovorus* JBD401. **d** Core genes between *L. acidophilus* 30SC and *L. amylovorus* JBD401. **e** Core genes between *L. acidophilus* FSI4 and *L. amylovorus* JBD401. **f** Core genes among five genomes; *L. acidophilus* NCFM, *L. acidophilus* La-14, *L. acidophilus* 30SC, *L. acidophilus* FSI4, and *L. amylovorus* JBD401. 1200 proteins were identified common to all of the strains along with unique genes of each genome; NCFM (662, 64.45%), La-14 (676, 63.97%), 30SC (837, 58.91%), FSI4 (558, 68.26%) and JBD401 (359, 76.97%)

Multiple genome alignment of *L. amylovorus* JBD401 with *L. acidophilus* NCFM, *L. acidophilus* 30SC, *L. acidophilus* FSI4, and *L. acidophilus* La-14 showed 55 super intervals in root alignment at the minimum weight of 15 (Fig. 4a). Overall GC content was 36.1%, and root alignment length was 2,713,521 bp. A total of 542 conserved regions were found among the five genomes by manual curation and were used for further analysis. Detailed deep analysis (COREGENES) [29] of the conserved region was executed to determine core proteins. Genes with a BLASTP threshold score of 75 were considered homologs in the analysis. Interestingly, 24 of the 1200 core genes corresponded to hypothetical proteins. A total of 662 proteins in *L. acidophilus* NCFM, 676 proteins in *L. acidophilus* La-14, 837 proteins in *L. acidophilus* 30SC, 558 proteins in *L. acidophilus* FSI4, and 359 proteins in *L. amylovorus* JBD401 were uniquely present in each genome (Fig. 4b ~ f Additional file 1: see supplementary results “Comparative genomics and functional analysis of *Lactobacillus amylovorus* JBD401”).

**Fig. 5:**
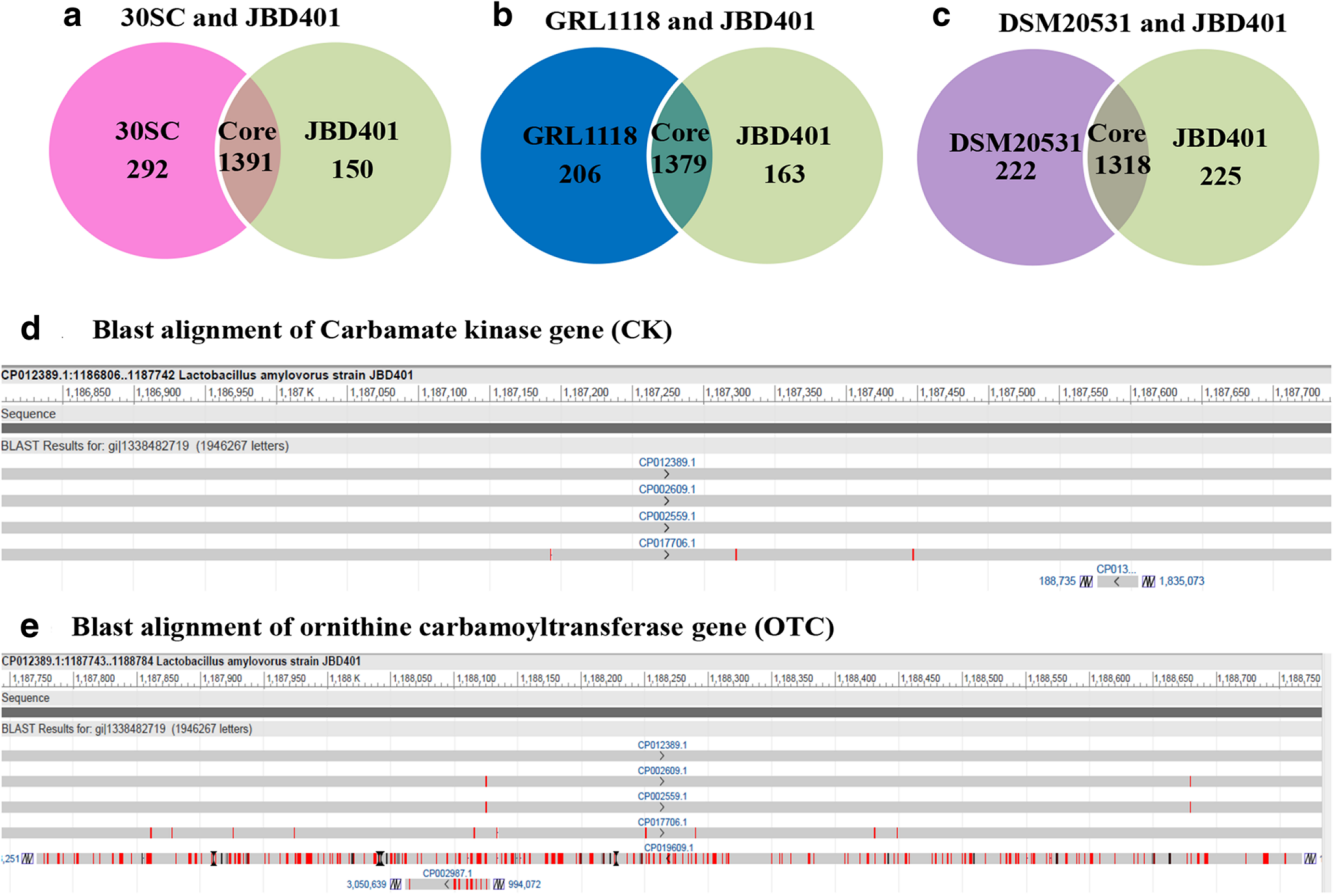
Comparative genome analysis of *Lactobacillus amylovorus* JBD401 with *Lactobacillus amylovorus* strains. **a** Core genes between *L. amylovorus* 30SC and *L. amylovorus* JBD401. **b** Core genes between *L. amylovorus* GRL1118 and *L. amylovorus* JBD401. **c** Core genes between *L. amylovorus* DSM20531 and *L. amylovorus* JBD401. **d** Blast alignment of carbamate kinase gene (1,186,806 bp–1,187,742 bp) of *L. amylovorus* JBD401. **e** Blast alignment of ornithine carbamoyltransferase gene (1,187,743 bp–1,188,784 bp) of *L. amylovorus* JBD401. **d**, **e** red bars in the alignment show mismatches and black bars show gaps

We further compared genes of *L. amylovorus* JBD401 with previously reported genomes of *L. amylovorus* strains in a pair-wise manner of group 30SC and JBD401, group GRL1118 and JBD401 and group DSM20531 and JBD401 (Fig. 5a, b, c). The numbers of core genes in [Group 30SC and JBD401] and in [GRL1118 and JBD401] were 1391 and 1379, respectively while that was only 1318 in [DSM20531 and JBD401]. The reduced number of core genes in [DSM20531 and JBD401] suggests that *L. amylovorus* DSM20531 is distant to *L. amylovorus* JBD401. We also performed additional BLAST searches to find the evidence where *L. amylovorus* JBD401 acquired genes of our interest from (Fig. 5d, e). Interestingly, BLAST results showed that CK (EC 2.7.2.2) was found in *L. amylovorus* JBD401 (100%), *L. amylovorus* GRL1118 (99.89%), *L. amylovorus* 30SC (99.89%), *L. amylovorus* DSM20531 (99.57%) and *Pyrodictium delaneyi* Su06 (100%) (Fig. 5d). OTC (EC 2.1.3.3) was found in *L. amylovorus* JBD401 (100%), *L. amylovorus* GRL1118 (99.71%), *L. amylovorus* 30SC (99.71%), *L. amylovorus* DSM20531 (98.84%), *Vagococcus penaei* CD276 (72.41%) and *Acetobacterium woodii* DSM1030 (83.33%) (Fig. 5e). BLAST alignment results suggest that CK and OTC genes are specific to *amylovorus* species in *Lactobacillus*. Rest of the important genes such as N-acyl-L-amino acid amidohydrolase (EC 3.5.1.14), N-acetyl-L,L-diaminopimelate aminotransferase (EC 2.6.1), glutamine synthetase type I (EC 6.3.1.2), aspartate aminotransferase (EC 2.6.1.1) and ornithine decarboxylase (EC 4.1.1.17) were also subjected to BLAST analysis to understand the regulation of biosynthetic pathways for various amino acids and polyamine metabolites in *L. amylovorus* starins (Additional file 1: Figure S3; Figure S4; Figure S5).

Functional annotation of *L. amylovorus* JBD401 was performed by the Blast2go (basic) tool [30]. Assigned controlled vocabularies or functional terms to the gene products were represented as a combined graph including summarized filtered pie charts (Additional file 1: Figure S6). Since the combined graph was too large for the presentation of functional data, it was divided into three categories: molecular function (MF), cellular component (CC), and biological process (BP). Annotation configuration was chosen with an e-value of 1e-4, which means that all sequences obtained with e-values higher than 1e-4 during blast were disregarded (Additional file 1: Figure S7).

### Identification of the complete set of genes for ammonia assimilation in *L. amylovorus* JBD401 to reduce blood levels

**Fig. 6:**
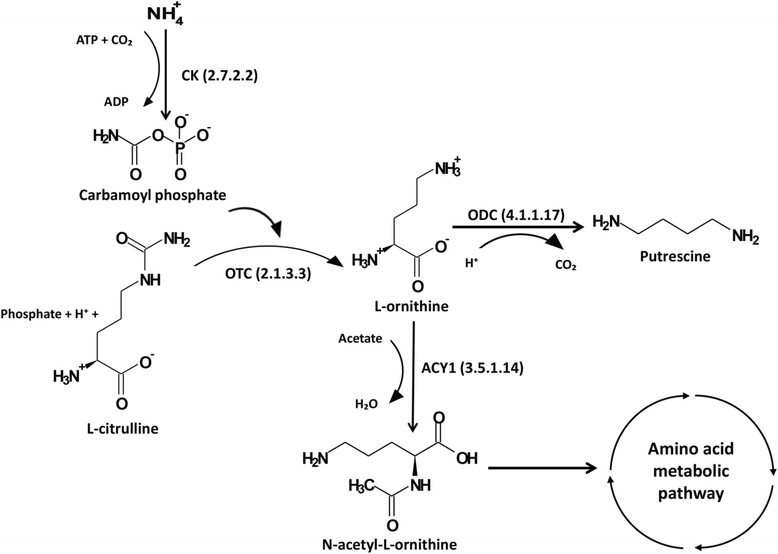
The metabolic pathways of *Lactobacillus amylovorus* JBD401 for ammonia assimilation. Gene annotation analysis revealed that *L. amylovorus* JBD401 had two very important genes, CK and OTC, for assimilation of ammonia. These two enzymes generate intermediate compounds for biosynthesis of various amino acids and polyamines

Comparative genome analysis showed that the genome of *L. amylovorus* JBD401 possessed the enzymes required for assimilation of ammonia: CK (EC 2.7.2.2) at AB283_06100 and OTC (EC 2.1.3.3) at AB283_06105. CK can synthesize carbamoyl phosphate from bicarbonate, ammonia, and ATP. The carbamoyl phosphate biosynthesized from ammonia can be converted into L-ornithine by OTC. L-ornithine is the key precursor metabolite for biosynthesis of various amino acids and polyamine compounds (Fig. 6). In addition to possession of the enzymes for biosynthesis of L-ornithine from ammonia, *L. amylovorus* JBD401 also possessed all of the enzymes for subsequent synthesis of various amino acids and polyamine compounds. This indicates that *L. amylovorus* JBD401 can actively absorb ammonia from its surrounding environment, i.e.*,* intestinal fluids, to assimilate ammonia into various amino acids and polyamine compounds. Active absorption of ammonia in intestinal fluids will reduce ammonia levels in intestinal fluids so that blood ammonia will traverse to intestinal fluids to reduce blood ammonia levels.

## Discussion

It is well known that many metabolic disturbances lead to hyperammonemia. Primary hyperammonemia is a genetic disease caused by mutation in the genes required for the urea cycle. There are six types of urea cycle disorder: N-acetylglutamate synthase deficiency; carbamoyl phosphate synthetase-1 deficiency; ornithine carbamoyltransferase deficiency; argininosuccinic acid synthetase deficiency (citrullinemia); argininosuccinase acid lyase deficiency (argininosuccinic aciduria); and arginase deficiency (argininemia) [31]. Primary hyperammonemia has recently attracted attention because of reports that an estimated 20% of cases of sudden infant death syndrome might to be due to an undiagnosed urea cycle disorder [32]. Secondary hyperammonemia is induced by metabolic defects characterized by reduced activity in enzymes that are not involved in the urea cycle, and various genetic or complex diseases such as propionic acidemia, methylmalonic acidemia, liver cirrhosis, or hepatic failure can induce secondary hyperammonemia [33]. Despite the fact that combined cases of primary and secondary hyperammonemia are frequently noted, there is no effective medication to treat hyperammonemia [34]. Considering recent advances that metabolic activities of gut microbiota in the intestine are associated with various diseases [35, 36], removal of ammonia by an intestinal bacterium represents a very promising approach for a therapeutic agent for hyperammonemia.

This work identified a *Lactobacillus* strain that effectively reduced blood ammonia levels in mice through the complete pathways for assimilation of ammonia. *L. amylovorus* JBD401 possessed two very important genes, CK and OTC. The enzymes encoded by these genes are required for assimilation of ammonia to synthesize L-ornithine, which is used as an intermediate compound for biosynthesis of various nitrogen-containing metabolites. *L. amylovorus* JBD401 also possessed the genes for N-acyl-L-amino acid amidohydrolase (EC 3.5.1.14) at AB283_1076 and N-acetyl-L, L-diaminopimelate aminotransferase (EC 2.6.1.) at AB283_0863 for biosynthesis of L-glutamate from L- ornithine. Gene annotation analyses showed that *L. amylovorus* JBD401 has a complete set of genes for bioconversion of L-glutamate to various amino acids, including glutamine synthetase type I (EC 6.3.1.2) at AB283_1841, aspartate aminotransferase (EC 2.6.1.1) at AB283_0491/0739/0740, N-acyl-L-amino acid amidohydrolase (EC 3.5.1.14) at AB283_1076, and N-acetyl-L,L-diaminopimelate aminotransferase (EC 2.6.1) at AB283_0863 (Fig. 6). *L. amylovorus* JBD401 also possesses the gene for ornithine decarboxylase (EC 4.1.1.17) at AB283_1019 for decarboxylation of L-ornithine to biosynthesize putrescine. Putrescine is a key intermediate metabolite for entry into the polyamine metabolic pathway. The presence of complete biosynthetic pathways for various amino acids and polyamine metabolites in the *L. amylovorus* JBD401 genome indicates that *L. amylovorus* JBD401 should actively absorb free amine from its surrounding environment for assimilation into various amino acids and polyamines. Assimilation of free amine into various amino acids and polyamines in the intestinal lumen of the host will reduce ammonia concentrations, resulting in traversing ammonia from the host body to the intestinal lumen to reduce blood ammonia levels.

Interestingly, the genome of *L. amylovorus* JBD401 contained 372 pseudogenes constituting 15.82% of the genome. Prokaryotic organisms usually have a very well organized single circular chromosome, which minimizes the content of intergenic nucleotide sequences. Generally *L. acidophilus* strains contain a small number of pseudogenes [22–25]. The higher number of pseudogenes in *L. amylovorus* JBD401 suggests that this strain might be in an active stage of evolution. During evolution process, *L. amylovorus* JBD401 seemed to acquire a complete set of genes to assimilate ammonia into various amino acids and polyamines.

The potential usefulness of probiotics in improving the symptoms of neurological diseases such as dementia, Reye syndrome, and Alzheimer’s disease is becoming a topic of great interest [37–39]. Especially, many epidemiological studies have repeatedly shown that higher adherence to Mediterranean diet was associated with lower risk of Alzheimer’s disease [40, 41]. Although the beneficial effects of probiotics on neurological diseases are becoming apparent recently, the underlying protective mechanism has remained unknown. Considering that ammonia is especially toxic to neurons [42], it would be logical to propose that a reduction of blood ammonia level has a beneficial effect in patients with neurological diseases. In this context, recent animal experiments showing that administration of *Lactobacillus* strains efficiently reduces blood ammonia levels have shed light on the question of how probiotics exert protective activity against neurological diseases.

Recent experiments clearly showed that administration of some *Lactobacillus* strains could be used to treat hyperammonemia patients [43–45]; however, their efficacies in reduction of blood ammonia levels were quite variable. In this study, we identified the best probiotic strain for treatment of hyperammonemia. Our work not only explains why *Lactobacillus* strains showed excellent effects in treatment of hyperammonemia, but also explains the mechanism of the *Lactobacillus*-assisted neuroprotective effect. In addition, this work will guide future screens to identify potential probiotic strains for treatment of hyperammonemia.

## Conclusions

High throughput screening of probiotic strains followed by subsequent animal experiments resulted in identification of an ideal strain for hyperammonemia, *L. amylovorus* JBD401. Comparative genomic analysis showed that *L. amylovorus* JBD401 had a complete set of genes required for ammonia assimilation into various amino acids and polyamines to reduce the blood ammonia levels of the host, providing an explanation for this effect of *L. amylovorus* JBD401 for hyperammonemia. The genome analysis confirmed that *L. amylovorus* JBD401 contains unusually large number of various pseudogenes suggesting an active stage of evolution which could explain the acquired functionality of ammonia assimilation. This work not only identifies the ideal strain but also explains the underlying mechanism on how a *Lactobacillus* strain could be used to treat hyperammonemia.

## Methods

### High throughput screening of ammonia-removing probiotic strains

The probiotic strains were obtained from the Gut Microbiota bank (https://www.gutmicrobiotabank.com) consisting of 23,689 strains. All strains were grown in De Mann Rogosa Sharpe (MRS) broth (Difco™) and maintained on MRS agar plates with routine sub-culturing at a regular interval of 15 days. For high throughput screening, the probiotic strains were first grown anaerobically in MRS broth at 37 °C until O.D. range from 0.45 to 1.0 at 600 nm, and 500 μL of each culture was transferred into 96 well plates. After addition of 10 μL of ammonium hydroxide (30 μg/mL) to the 96 well plates, the cultures were incubated anaerobically at 37 °C for 30 min and then centrifuged at 4000 rpm for 15 min at 4 °C. After centrifugation, the supernatants in the 96 well plates were transferred into new 96 well plates, and the ammonia concentrations in each supernatant were measured by an ammonia assay kit (AA0100; Sigma-Aldrich) according to manufacturer’s protocol.

### Measuring blood and brain ammonia levels and caspase induction in test animals

*In vitro* high throughput screening resulted in identification of *L. amylovorus* JBD401 removing free ammonia in its surrounding MRS medium. To evaluate the efficacy of *L. amylovorus* JBD401 to hyperammonemia, 1 × 10^7^ CFU of *L. amylovorus* JBD401 culture was orally administered to four-week-old male ICR mice for 7 days. Hyperammonemia was induced by intravenous administration of 100 mg/kg of NH_4_Cl into the tail on the last day. Fifty minutes after injection of ammonia, blood was collected, and the brain was removed. Brain tissue collection procedure was initiated after animals had been euthanized by cervical dislocation. All efforts were made to minimize suffering. The blood was centrifuged to collect supernatant serum for measurement of ammonia. The brains were ground using a homogenizer, and the supernatants of the ground brains were used to quantitate ammonia concentrations. The ammonia concentrations in each supernatant samples were measured by an ammonia assay kit (AA0100; Sigma-Aldrich) according to manufacturer’s protocol at 200–300 nm using a nanodrop machine (Thermo Scientific).

Caspase induction was measured using a Fluorescent-Labeled Inhibitor of Caspases (FLICA™) assay kit. Brain tissue of mouse was sliced using a cryotome (Thermo Scientific). The FLICA Caspase 9 reagent was reconstituted with 50 μL of DMSO to form the stock concentrate, which was diluted with 200 μL PBS to give the working solution. The working solution was directly added to samples and controls at a ratio of 1:30 to 1:60 and incubated for 15 to 45 min. Cells were washed and centrifuged two or three times. Cells were labeled with Hoechst stain or DAPI and/or fixed with fixative included in the assay kit. Data were analyzed using a fluorescence microscope (Nikon).

### Genome sequencing and assembly

The *L. amylovorus* JBD401 (KCTC 11515BP) genome was sequenced by next-generation sequencing using 454 (454 GS FLX) and Illumina methods. Initially, 454 DNA reads were assembled into contigs with the PHRED/PHRAP/CONSED [46] software package and subcontracted to Macrogen Inc. Further contigs were assembled using Geneious pro.5.5 software [47] and CLC workbench software (CLC Bio-Qiagen). Mapping of assembled contigs was performed by Projector 2 software [48]. Gaps were closed by adopting a PCR-based strategy. Ends of scaffolds were used for primer design, and the inter-scaffold region was amplified by sequencing multiplex PCR products using a long-range PCR kit (Qiagen). Primer3 software [49] was used to design primers, and primer synthesis and PCR product sequencing were performed at Xenotech.

### Genome annotation

The complete genome sequence was subjected to an automated annotation process by RAST [50]. Quality of reads was examined by FastQC [51] (Additional file 1: Figure S2), and contigs were ordered against a reference, *Lactobacillus amylovorus* GRL1118, using Mauve (multiple genome alignment) (Additional file 1: Figure S1) [52]. According to the RAST server, the genome sequence of *L. amylovorus* JBD401 contains 230 subsystems. Every subsystem is a collection of functional roles and associated to each other in a system, for example, a metabolic pathway system. The genome was further curated manually using web-based software and databases. FrameD [53] was used for prediction of ORFs, and their starts were manually adjusted based on blast alignment. The gene model was predicted by Glimmer [54] and compared with the following public databases: COGs [55], Interpro [56], Pfam [57], Prints [58], PROSITE [59], Smart [60], Swissprot [61], and Tigrfams [62]. Tblastx [63] was used to reanalyze intergenic regions for ORFs. Hmmer [64] on pfam 5.4 was used for motif analysis. Stringent parameters were used to identify tRNAs by trnascan-se [65] and ribosomal binding sites by RBSfinder. After resequencing, the Genome atlas was generated by Geneious 5.5 and PATRIC [66]. Prophage regions within the genome were identified using PHAST. Whole-genome nucleotide alignments were generated and visualized by Mauve. Identification of core genes and unique genes was carried out by coregenes 3.0.

### *In silico* analysis of antibiotic resistant genes in genome of *L. amylovorus* JBD401

The presence of homologs of various antibiotic resistance genes in the genome of *L. amylovorus* JBD401 was checked by performing BLASTp searches of the entire genome (under multiple gene annotation category) against the ARDB database [67]. We used identity threshold of 40% which is also a default search criteria in ARDB database. Similarly, a previous study have also used relaxed identity threshold of 35% to identify antibiotic resistance genes in swine gut metagenomes [68].

### Functional analysis

Gene ontology (GO) analysis was carried out using Blast2GO basic account to analyze the FASTA-formatted sequences of predicted genes of *L. amylovorus* JBD401 genome into the three GO categories of biological process, molecular function, and cellular component. Protein sequences of *L. amylovorus* JBD401 were annotated with blastp followed by Blast2GO mapping and GO annotation. This provided vocabularies from GO terms, enzyme codes (EC), InterPro IDs, and KEGG pathways. Homologous sequences were identified by blast and mapped to GO terms that were associated with blast hits. Mapping of GO terms was reduced by GO-slim ontology. Multiple testing in Blast2GO was performed by Fisher’s exact test to compute GO significance levels.

## Additional file

Additional file 1: Annotation and organization of *L. amylovorus* JBD401 genome in this study. (DOCX 4200 kb)

## Abbreviations

A: Aspartic
ANI: Average nucleotide identity
Asp: Aspartic acid
BP: Biological process
C: Cysteine
CC: Cellular component
CK: Carbamate kinase
Cys: Cysteine
EC: Enzyme codes
FLICA™: Fluorescent-labeled inhibitor of caspases
FOS: Fructooligosaccharides
Gln: Glutamine
Glu: Glutamic acid
Gly: Glycine
GO: Gene ontology
HGT: horizontal gene transfer
*L.*: *Lactobacillus*
M: Metallo
MF: Molecular function
MRS: De Mann Rogosa Sharpe
OTC: Ornithine carbamoyltransferase
RhBG: (Rh family, B glycoprotein)
RhCG: (Rh family, C glycoprotein)
S: Serine
Ser: Serine
T: Threonine
Thr: Threonine
VTEC: verocytotoxin-producing *Escherichia coli*
