# Supplementary material for: Elucidation of the anti-hyperammonemic mechanism of Lactobacillus amylovorus JBD401 by comparative genomic analysis
Source: BMC Genomics. 2018 Apr 25;19:292. doi: 10.1186/s12864-018-4672-3 (PMC5918772; doi:10.1186/s12864-018-4672-3)
Supplement: Supplementary file 1 — Annotation and organization of L. amylovorus JBD401 genome in this study. (DOCX 4200 kb) [file 12864_2018_4672_MOESM1_ESM.docx]

**Additional file 1**

**Elucidation of the anti-hyperammonemic mechanism of *Lactobacillus amylovorus* JBD401 by comparative genomic analysis**

*Parul Singh^1,4^, Hea-Jong Chung^1,4^, In-Ah Lee ^3^, Roshan D’Souza^1^, Hyeon-Jin Kim^2^ and Seong-Tshool Hong^1, *^*

^1^Department of Biomedical Sciences and Institute for Medical Science, Chonbuk National University Medical School, Jeonju, Chonbuk 54907, South Korea.

^2^JINIS BDRD institute, JINIS Biopharmaceuticals Co., 948-9 Dunsan, Bongdong, Wanju, Chonbuk 55321, South Korea.

^3^Current address: Department of Chemistry, Gunsan National University, Gunsan, Chonbuk 51450, South Korea.

^4^These authors contributed equally to this work.

^*^Corresponding authors: Seong-Tshool Hong (e-mail: seonghong@chonbuk.ac.kr)

**Additional file 1: Supplementary Results**

*Genome features of Lactobacillus amylovorus* JBD401

Bacteria require a lot of energy to synthesize amino acids from scratch. Many pathways begin with central metabolites using common compounds for synthesis. *L. amylovorus* JBD401 appears to be unable to synthesize seven amino acids—valine, leucine, isoleucine, histidine, tyrosine, phenylalanine, and tryptophan due to the absence of a complete biosynthetic pathway. However, a conversion pathway is available for another seven amino acids—alanine, methionine, lysine, arginine, glutamic acid, asparagine, and proline. It appears that *L. amylovorus* JBD401 possesses a partial citrate cycle due to the presence of fumarate hydratase (*fum*C, AB283_0917) and succinate dehydrogenase (*SDH1*, AB283_1112), which produce malate, fumarate, and succinate.

Bacteriocin helveticin J is a heat-sensitive and partially purified bacteriocin with a limited range of antagonistic activity. This activity functions in the production of many active substances that interfere with the growth of some anaerobic gastrointestinal tract pathogens in the same ecological niche, such as *Helicobacter* *pylori* and *Clostridium difficile*. Helveticin J is large, with a molecular weight of 37 kDa, and is closely related to caseicin 80 and acidophilucin A, but differs from Lactacins B and Lactacins F. The bacteriocin ABC-transporter auxiliary protein AB283_2114 plays an important role during transportation and has two regions, one of which is hydrophobic, and the other is a carbon region or ATP-binding region. The exact role of this protein has not been identified, but it has potential proteolytic activity due to the presence of one N-terminal extension (approximately 150 amino acids) that breaks the double-glycine leader of pre-peptides.

*Comparative genomics and functional analysis of Lactobacillus amylovorus* JBD401

To reveal more extensive details from a broader sampling of the species, genes of *L. amylovorus* JBD401 were individually compared with previously reported genomes of *L. acidophilus* strains in a pair-wise manner of group NCFM and JBD401, group La-14 and JBD401, group 30SC and JBD401 and group FSI4 and JBD401 (Fig. 4b, c, d, e of main document). First, core proteins were defined in each group together with unique genes by COREGENES. The number of homologous proteins in each group was almost identical for group NCFM and JBD401 (1259), group La-14 and JBD401 (1271), and group FSI4 and JBD401 (1257) but not for group 30SC and JBD401 (1396). A larger shared core suggests that *L. acidophilus* 30SC is more closely related to *L. amylovorus* JBD401. The number of unique proteins in each group related to *L. amylovorus* JBD401 was also correlated with group NCFM and JBD401 (NCFM: 300), group La-14 and JBD401 (La-14: 288), group 30SC and JBD401 (30SC: 163), and group FSI4 and JBD401 (FSI4: 302), containing the largest number of unique proteins. This suggests that many genes unique to each genome might have been acquired by horizontal gene transfer.

**Additional file 1: Figure S1**

**
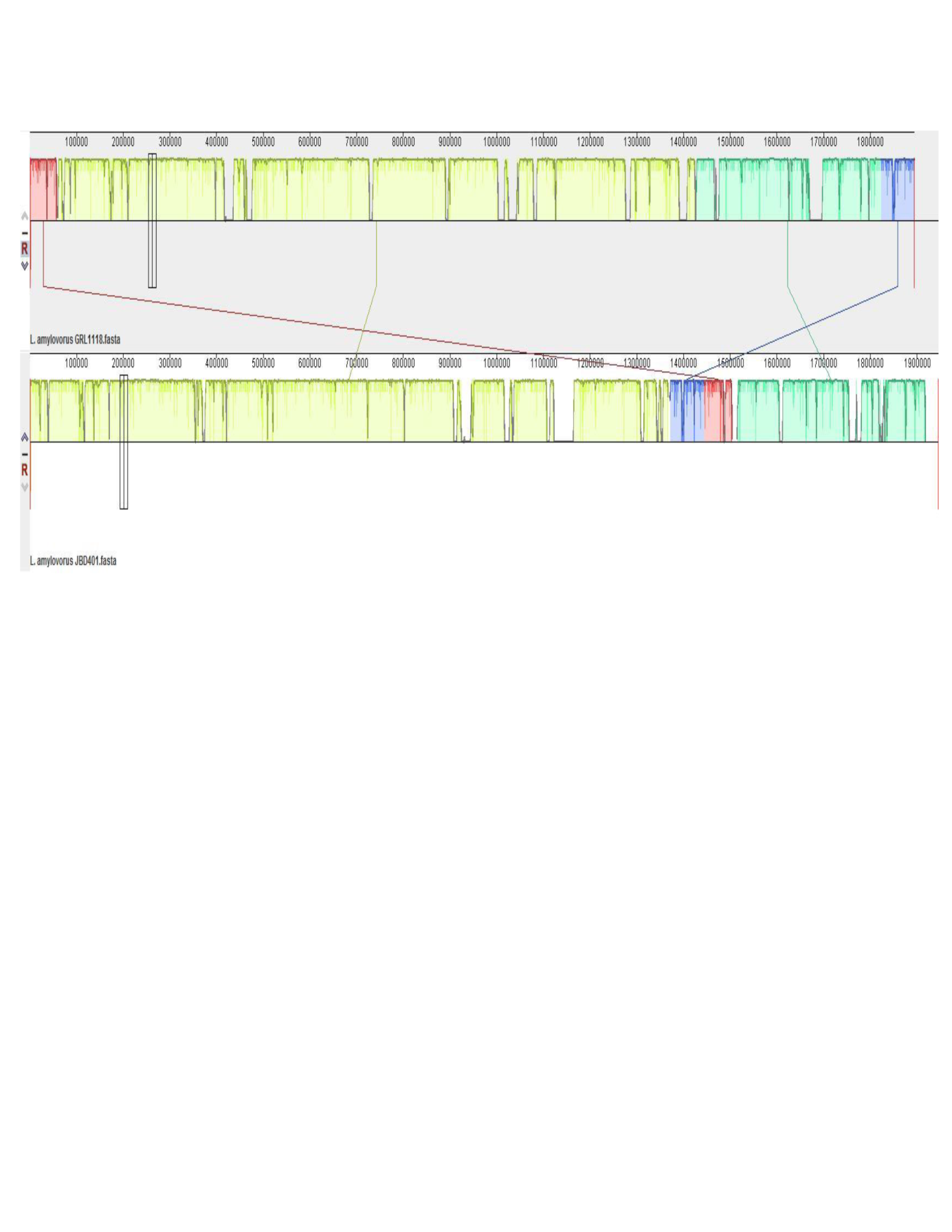
**

**Figure S1: Draft genome of *Lactobacillus*** *amylovorus* **JBD401 was ordered against *Lactobacillus amylovorus* GRL1118.** The overall arrangement of the draft genome of *L. amylovorus* JBD401 was done using progressive Mauve algorithm and aligned to the reference, *Lactobacillus amylovorus* GRL1118. The method of ordering produced a well ordered draft with only few major gaps. These gaps were not aligned and probably contain sequence elements specific to individual genome. Root alignment showed 6 super intervals and root alignment length was 2,136,631. Organisms showed 38.1% GC.

**Additional file 1: Figure S2**

**
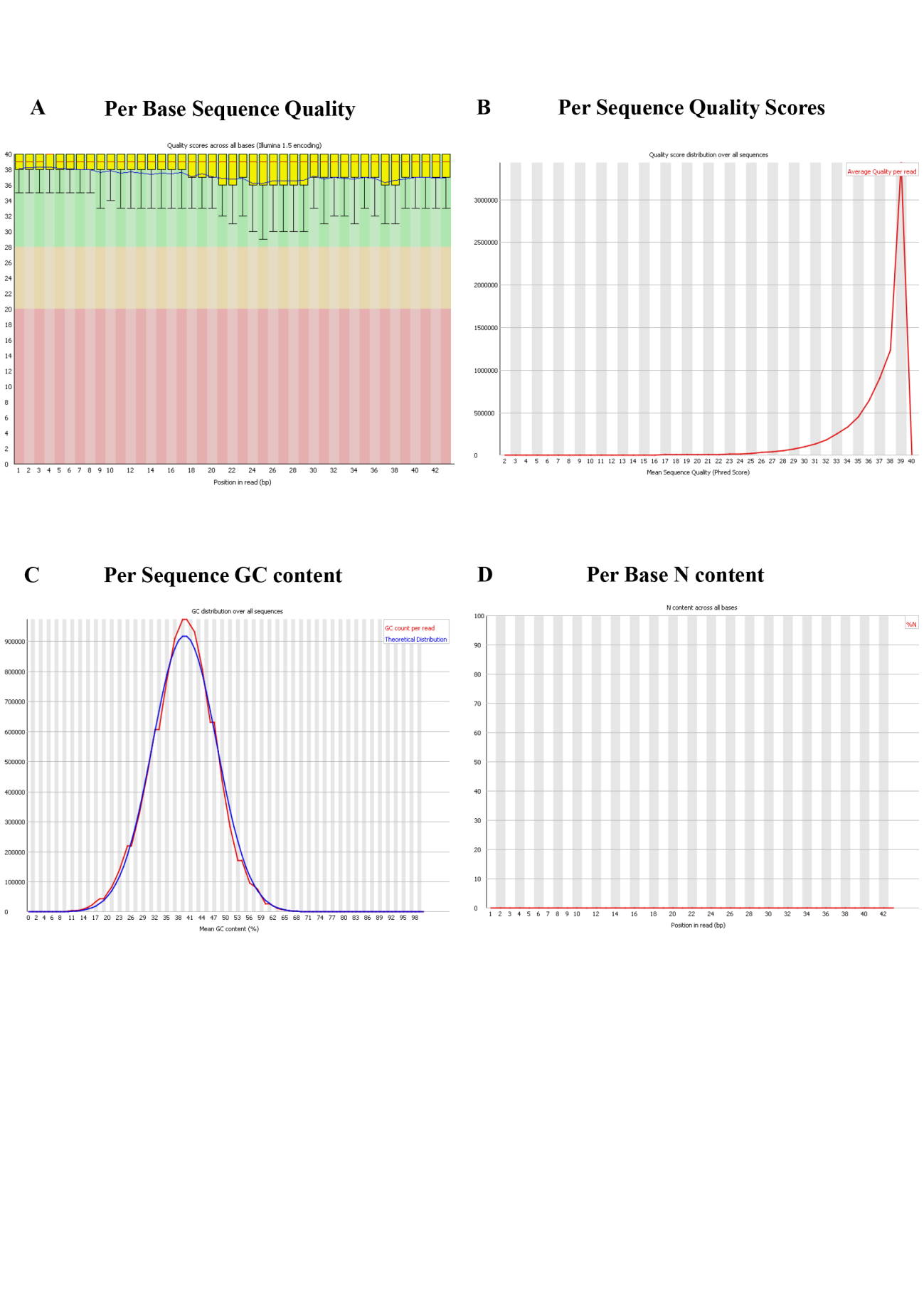
**

**Figure S2: Quality assessment report of *Lactobacillus*** *amylovorus* **JBD401 sequencing (illumina reads) by FastQC. (A)** Per base sequence quality graph of *L. amylovorus* JBD401. This graph shows a BoxWhisker plot is present for each position. Plot has four elements: central red line (shows median value), yellow boxes (shows inter-quartile range, 25-75%), Upper and lower whiskers (shows 10% and 90% points) and blue line (shows mean quality). Considering all these parameters The Y-axis on the graph represents per base sequence quality score. Colour of the background of the graph plays important role to determine the quality of sequencing calls: good quality (green), average quality (orange) and poor quality (red). Here, all reads are present on green colour background which ensures very good quality of reads. **(B)** Per sequence quality scores of *L. amylovorus* JBD401. This graph shows whether a subset of sequences has universally low quality. If many subsets of sequences have low quality then it indicates systematic problem. Here, a significant proportion of the run is showing good quality reads because most frequently observed mean quality is below 27. **(C)** Per sequence GC content graph of *L. amylovorus* JBD401. This graph shows computation of the GC content across the whole length of each sequence and further compares it to a modeled normal distribution of GC content. Here, actual distribution (red) coincides to the expected distribution (blue), a central peak which corresponds to the overall GC content of the underlying genome. This graph is considered good because less than 15% of reads are represented by sum of deviations from the normal distribution. **(D)** Per base N content graph of *L. amylovorus* JBD401. This graph measures the percentage of base calls at each position for which an N was called. Here, graph is linear and position of N content is 0% which shows that sequencer was able to make base call effectively.

**Additional file 1: Figure S3**

**
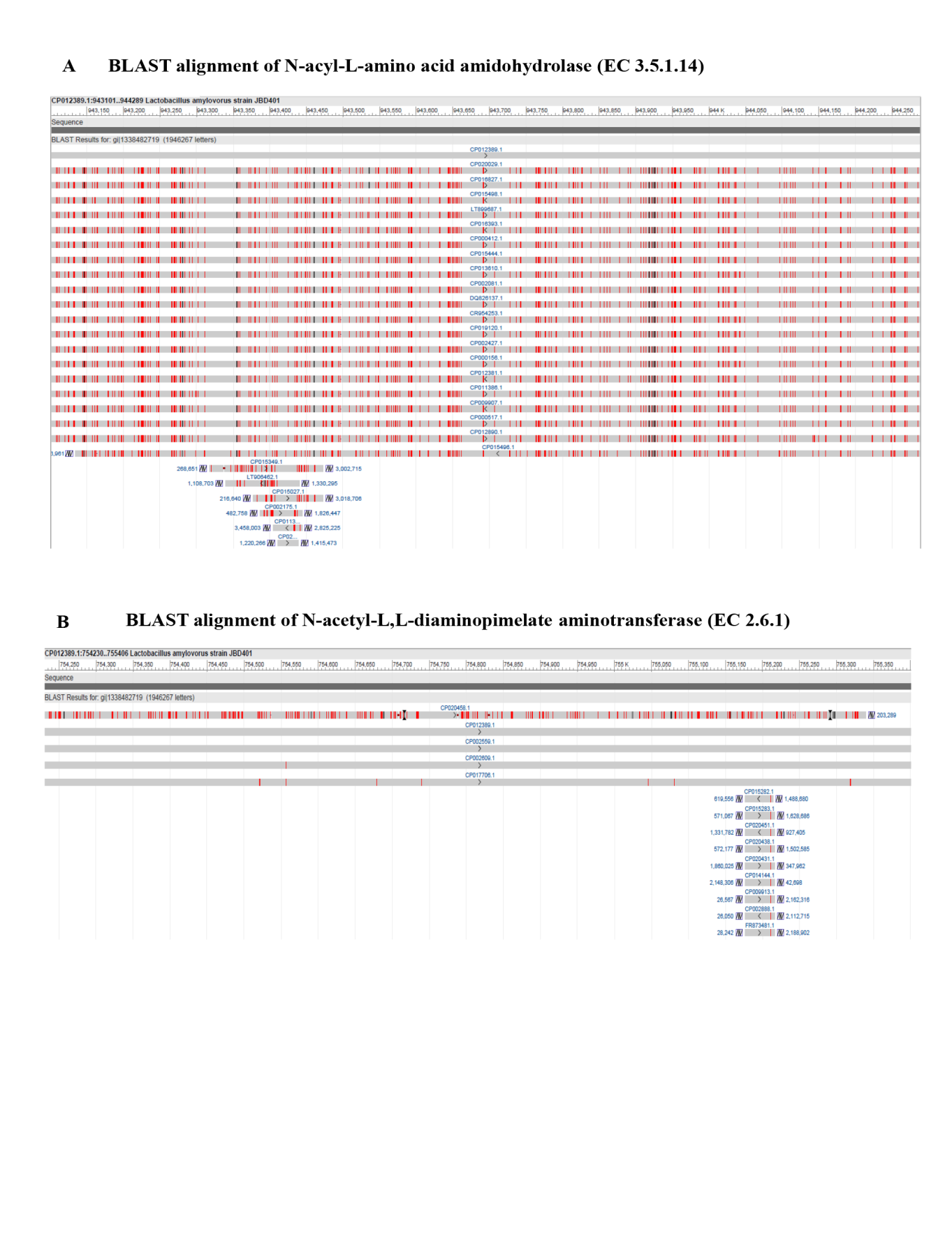
**

**Figure S3: BLAST alignment of genes of *Lactobacillus amylovorus* JBD401 involved in biosynthesis of L-glutamate from L- ornithine. (A)** Blast alignment of N-acyl-L-amino acid amidohydrolase gene (943101bp-944289bp) of *L. amylovorus* JBD401. Total 27 sequences produced significant alignments with 77% to 100% identity. Most of the predicted sequences belonged to strains of *L. helvetics* and *L. delbrueckii.* Interestingly, no amylovorus strains produced alignment except *L. amylovorus* JBD401. **(B)** Blast alignment of N-acetyl-L,L-diaminopimelate aminotransferase gene (754230bp-755406bp) of *L. amylovorus* JBD401. Total 14 sequences produced significant alignments with 74.5% to 100% identity. *L. amylovorus* JBD401, *L. amylovorus* 30SC, *L. amylovorus* GRL1118 and *L. amylovorus* DSM20531 produced alignment with 100%, 99.91%, 99.83% and 99.23% percent identity respectively. **(A,B)** red bars in the alignment show mismatches and black bars show gaps.

**Additional file 1: Figure S4**

**
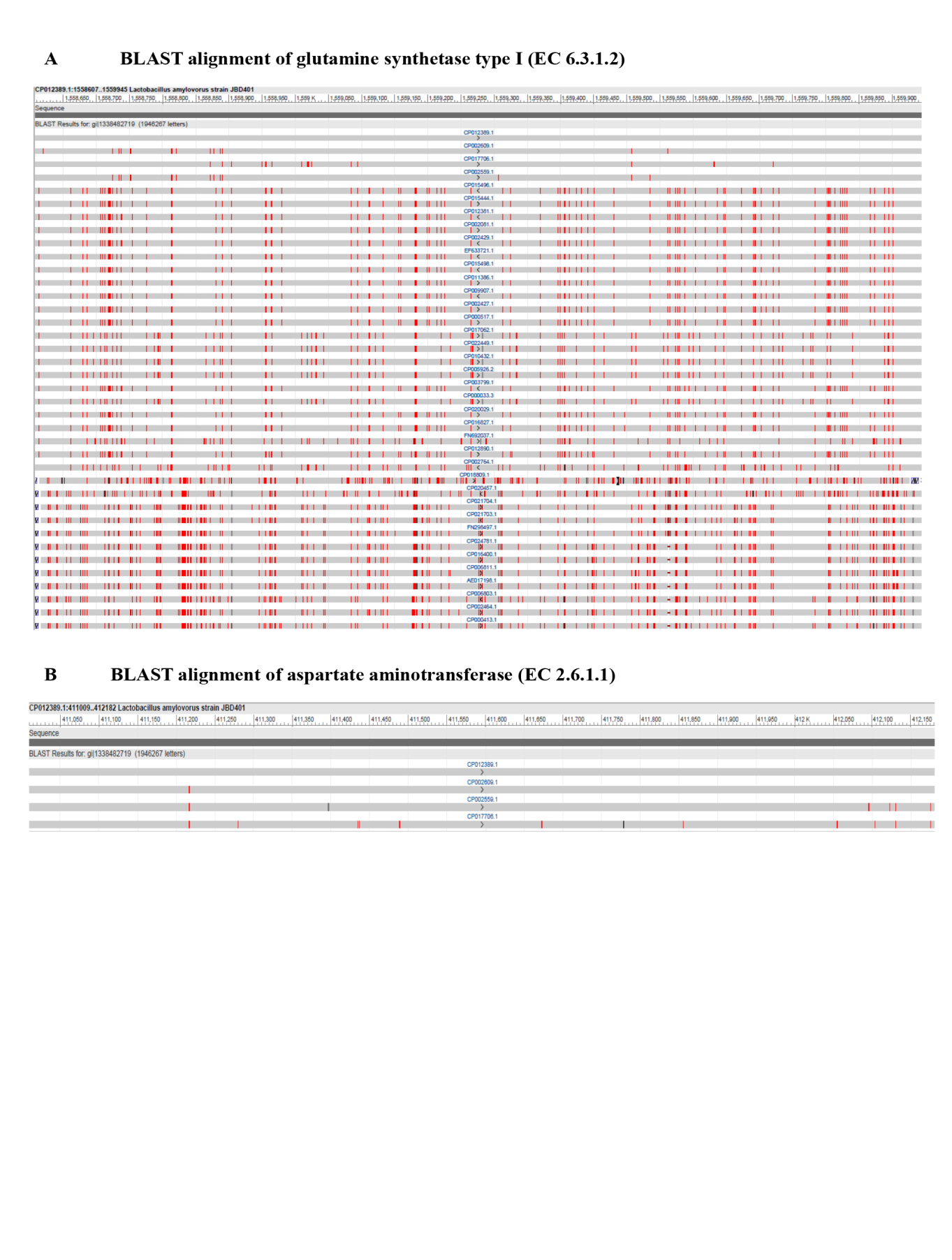
**

**Figure S4: BLAST alignment of genes of *Lactobacillus amylovorus* JBD401 involved in bioconversion of L-glutamate to L-glutamine and L-aspartate. (A)** Blast alignment of glutamine synthetase type I gene (1558607bp-1559945bp) of *L. amylovorus* JBD401. Total 38 sequences produced significant alignments with 77.8% to 100% identity. Most of the predicted sequences belonged to strains of *L. amylovorus*, *L. helvetics*, *L. acidophilus* and *L. johnsonii.* **(B)** Blast alignment of aspartate aminotransferase gene (411009bp-412182bp) of *L. amylovorus* JBD401. Interestingly, all four predicted sequences belonged to only amylovorus strains. *L. amylovorus* JBD401, *L. amylovorus* GRL1118, *L. amylovorus* 30SC and *L. amylovorus* DSM20531 produced alignment with 100%, 99.91%, 99.48% and 98.72% percent identity respectively. **(A,B)** red bars in the alignment show mismatches and black bars show gaps.

**Additional file 1: Figure S5**

**
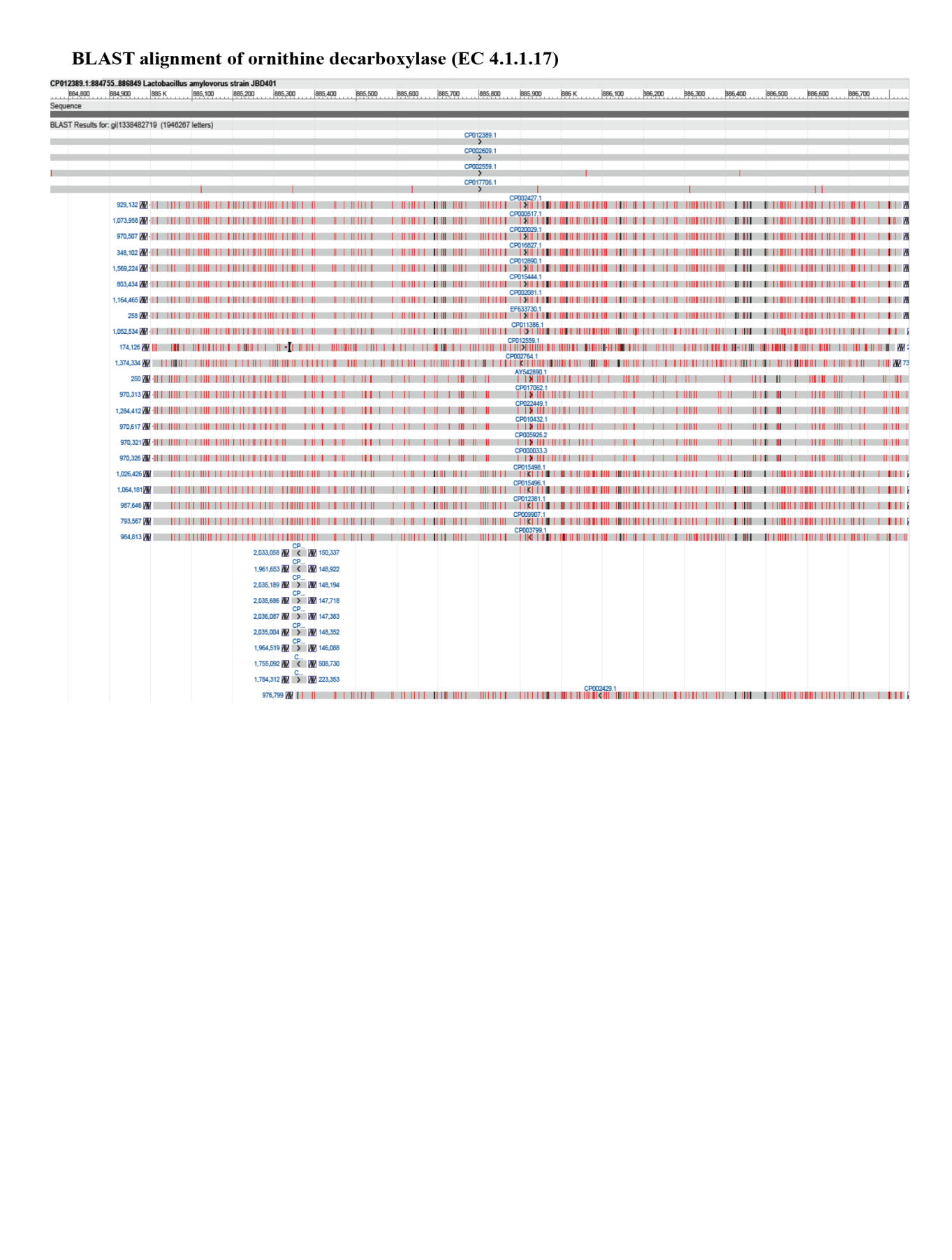
**

**Figure S5: BLAST alignment of gene of *Lactobacillus amylovorus* JBD401 involved in decarboxylation of L-ornithine.** Blast alignment of ornithine decarboxylase gene (884755bp-886849bp) of *L. amylovorus* JBD401. Total 36 sequences produced significant alignments with 74% to 100% identity. Most of the predicted sequences belonged to strains of *L. amylovorus*, *L. helvetics*, *L. acidophilus* and *Histophilus somni.* *L. amylovorus* JBD401, *L. amylovorus* GRL1118, *L. amylovorus* 30SC and *L. amylovorus* DSM20531 produced alignment with 100%, 99.80%, 99.71% and 99.23% percent identity respectively. Red bars in the alignment show mismatches and black bars show gaps.

**Additional file 1: Figure S6**

**
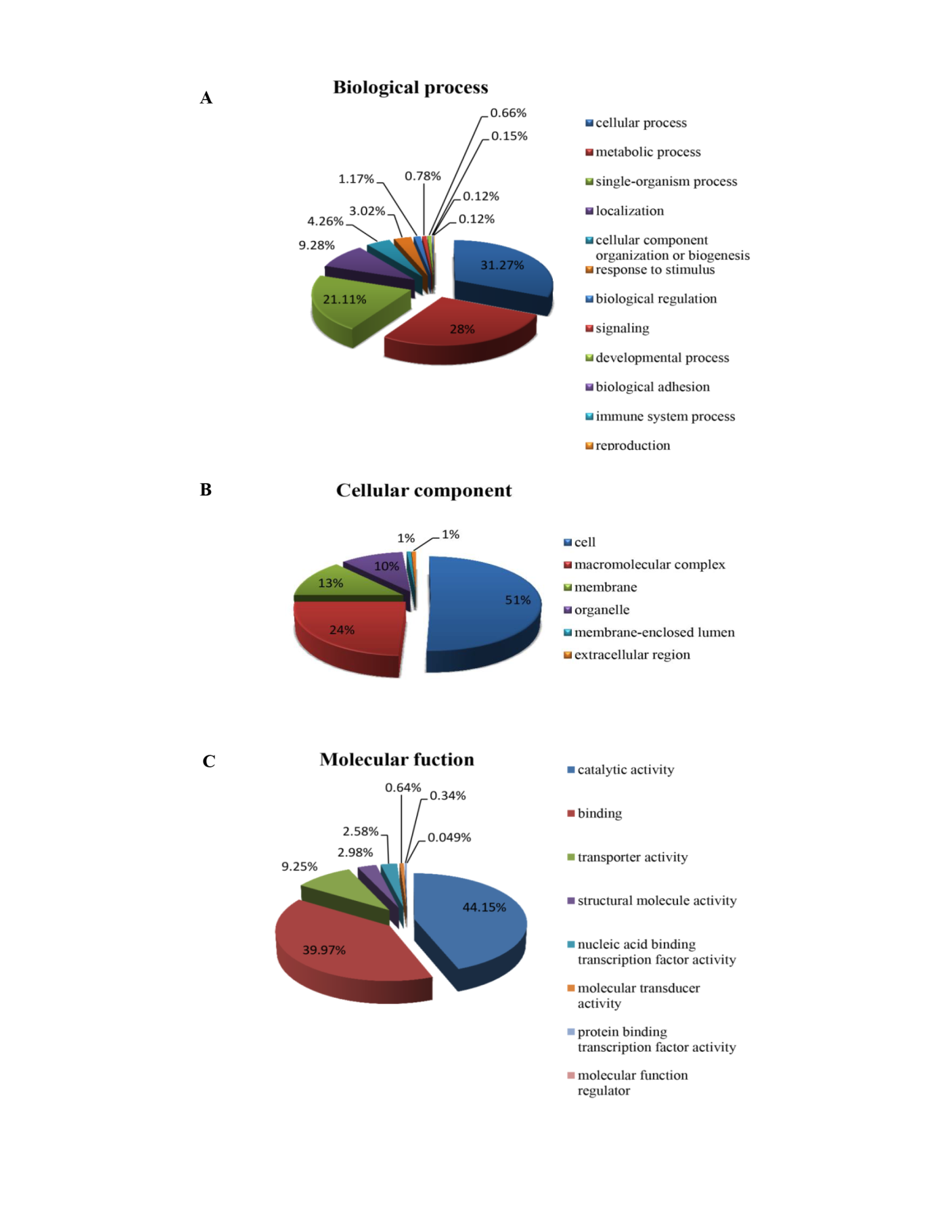
**

**Figure S6: Functional genome analysis of *Lactobacillus*** *amylovorus* **JBD401. (A)** The Pie chart of biological process of *L. amylovorus* JBD401 shows relative abundance of different categories where 31.27% annotation were related to cellular process, 28% to metabolic process, 21.11% to single-organism process, 9.28% to localization, and 4.26% to cellular component organization. **(B)** Pie chart of cellular component of *L. amylovorus* JBD401. The cellular component represents all major components from macromolecular complex (24%) and membrane (13%) to organelle (10%) and cell (51%). **(C)** Pie chart of molecular function of *L. amylovorus* JBD401. Common molecular functions of this genome are catalytic activity (44.15%) and binding proteins or nucleic acids (39.97%) followed by transporter activity (9.25%) and structural molecule activity (2.98%).

**Additional file 1: Figure S7**


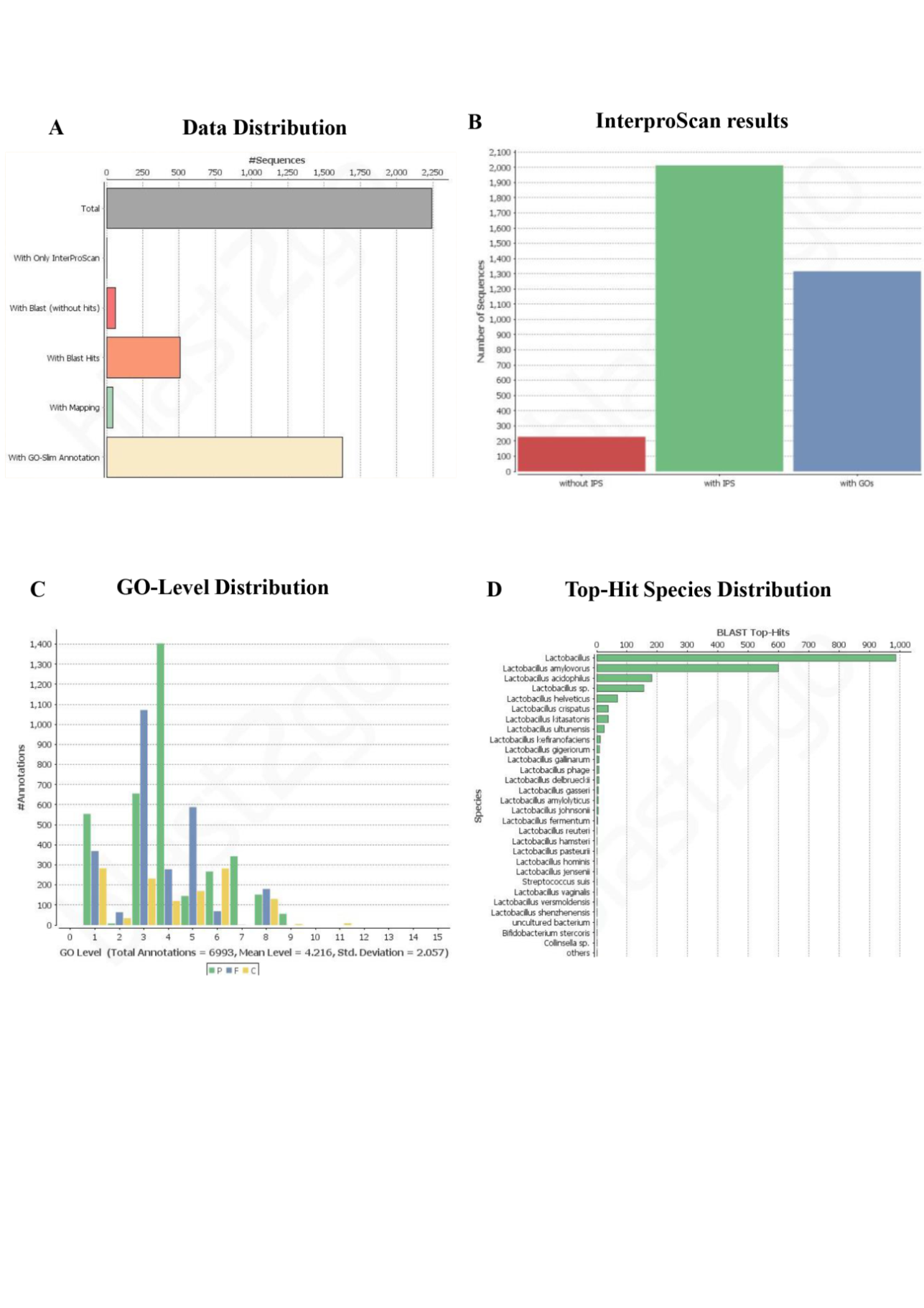


**Figure S7: Functional annotation of *Lactobacillus*** *amylovorus* **JBD401.** **(A)** Result of data distribution process of *L. amylovorus* JBD401. Total 2,242 protein sequences were input, among them 2 sequences were with only InterProScan hits, 63 sequences were without Blast hits, 509 sequences were with Blast hits, 43 sequences were with Mapping and 1625 sequences were with GO-Slim annotation. **(B)** InterProScan result of *L. amylovorus* JBD401. 89.83% were mapped with InterProScan ID and 65.39% of them were linked with GO term mapping. **(C)** Chart of GO level distribution for *L. amylovorus* JBD401. This bar chart shows all GO terms for all 3 categories P (green)=Biological process, F (blue)=Molecular function, C (yellow)=Cellular component for a given GO level taking into account the GO hierarchy. Overall, 72.47% of the input sequences were assigned functionally with a total of 6993 GO terms. Mean GO level i.e. distance between ontology root term and GO term is 4.216 with 2.057 standard deviation. **(D)** Top hit species distribution chart of *L. amylovorus* JBD401. It indicates species with higher similarity score in blast hits against *L. amylovorus* JBD401 such as *Lactobacillus* (986), *L. amylovorus* (599), *L. acidophilus* (182), *Lactobacillus* sp. (155), *L. helveticus* (69) etc.

**Additional file 1:** **Table S1: List of proteases/peptidases related transporters in *Lactobacillus*** *amylovorus* **JBD401.**

| **Location** | **Function** |
| --- | --- |
| AB283_0170 | Oligopeptide ABC transporter, periplasmic oligopeptide-binding protein OppA |
| AB283_0171 | Oligopeptide ABC transporter, periplasmic oligopeptide-binding protein OppA |
| AB283_0173 | Oligopeptide ABC transporter, periplasmic oligopeptide-binding protein OppB |
| AB283_0174 | Oligopeptide transport system permease protein OppC |
| AB283_0175 | Oligopeptide transport ATP-binding protein OppD |
| AB283_0176 | Oligopeptide transport ATP-binding protein OppD |
| AB283_0177 | Oligopeptide transport ATP-binding protein OppF |
| AB283_0505 | Oligopeptide ABC transporter, periplasmic oligopeptide-binding protein OppA |
| AB283_0506 | Oligopeptide ABC transporter, periplasmic oligopeptide-binding protein OppA |
| AB283_1380 | Oligopeptide ABC transporter, periplasmic oligopeptide-binding protein OppA |
| AB283_1381 | Oligopeptide ABC transporter, periplasmic oligopeptide-binding protein OppA |
| AB283_1382 | Oligopeptide ABC transporter, periplasmic oligopeptide-binding protein OppA |
| AB283_1478 | Oligopeptide ABC transporter, periplasmic oligopeptide-binding protein OppA |
| AB283_1479 | Oligopeptide ABC transporter, periplasmic oligopeptide-binding protein OppA |
| AB283_1480 | Oligopeptide transport system permease protein OppC |
| AB283_1481 | Oligopeptide transport system permease protein OppB |
| AB283_1482 | Oligopeptide transport ATP-binding protein OppF |
| AB283_1483 | Oligopeptide transport ATP-binding protein OppD |
| AB283_1568 | Oligopeptide ABC transporter, periplasmic oligopeptide-binding protein OppA |
| AB283_1569 | Oligopeptide ABC transporter, periplasmic oligopeptide-binding protein OppA |
| AB283_1584 | Oligopeptide ABC transporter, periplasmic oligopeptide-binding protein OppA |
| AB283_1585 | Oligopeptide ABC transporter, periplasmic oligopeptide-binding protein OppA |
| AB283_1586 | Oligopeptide ABC transporter, periplasmic oligopeptide-binding protein OppA |
| AB283_1671 | Oligopeptide ABC transporter, periplasmic oligopeptide-binding protein OppA |
| AB283_1781 | Oligopeptide ABC transporter, periplasmic oligopeptide-binding protein OppA |
| AB283_1782 | Oligopeptide ABC transporter, periplasmic oligopeptide-binding protein OppA |
| AB283_2000 | Oligopeptide ABC transporter, periplasmic oligopeptide-binding protein OppA |

**Additional file 1:** **Table S2: List of proteases/peptidases in *Lactobacillus*** *amylovorus* **JBD401 genome.**

| **Proteases/peptidases** | **Family** | **Location** |
| --- | --- | --- |
| Serine protease, DegP/HtrA, do-like (EC 3.4.21.-) | S1C | AB283_0043 |
| Alpha/beta hydrolase fold (EC 3.8.1.5) | S33 | AB283_0047,AB283_0089, AB283_1520, AB283_1992, AB283_2158 |
| Heat shock protein HtpX (EC 3.4.24.-) | M48B | AB283_0056 |
| Lipase/esterase | S9C | AB283_0105, AB283_0698, AB283_1524, AB283_1963 |
| N-acetylglucosamine-6-phosphate deacetylase (EC 3.5.1.25) | M38 | AB283_0107 |
| Asparagine synthetase [glutamine-hydrolyzing] (EC 6.3.5.4) | C44 | AB283_0122 |
| endopeptidase O | M13 | AB283_0131, AB283_0132, AB283_1175, AB283_1448 |
| Pyrrolidone-carboxylate peptidase (EC 3.4.19.3) | C15 | AB283_0157 |
| Aminopeptidase C (EC 3.4.22.40) | C1B | AB283_0167, AB283_0168, AB283_0178, AB283_0317, AB283_0922 |
| FIG00745161: hypothetical protein | M79 | AB283_0181 |
| CTP synthase (EC 6.3.4.2) | C26 | AB283_0210 |
| Dipeptidase (EC 3.4.-.-) | C69 | AB283_0212, AB283_1471, AB283_1730, AB283_1980, AB283_2153 |
| GMP synthase (EC 6.3.5.2) | C26 | AB283_0222 |
| FtsH peptidase (EC 3.4.24.-) | M41 | AB283_0252 |
| ComC peptidase | A24A | AB283_0264 |
| DNA repair protein RadA peptidase | S16 | AB283_0320 |
| Xaa-Pro dipeptidase (EC 3.4.13.9) | M24B | AB283_0417 |
| Glucosamine-fructose-6-phosphate aminotransferase (EC 2.6.1.16) | C44 | AB283_0444 |
| Methionine aminopeptidase (EC 3.4.11.18) | M24A | AB283_0606 |
| Hydrolases of the alpha/beta superfamily | S9C | AB283_0613, AB283_0723 |
| FIG009210: peptidase | M16B | AB283_0646 |
| ATP-dependent Clp protease (EC 3.4.21.92) | S14 | AB283_0684 |
| hypothetical protein | S9C | AB283_0699 |
| Esterase | S12 | AB283_0753 |
| N-acetyl-L,L-diaminopimelate deacetylase (EC 3.5.1.47) | M20D | AB283_0860, AB283_1076 |
| Beta-lactamase class C, Penicillin-binding protein | S12 | AB283_0866, AB283_1027, AB283_1631, AB283_1768, AB283_1811, AB283_ 2236 |
| Choloylglycine hydrolase (EC 3.5.1.24) | C59 | AB283_0907, AB283_1061, AB283_1133 |
| ATP-dependent protease HslV (EC 3.4.25.-) | T1B | AB283_1005 |
| Xaa-His dipeptidase (EC 3.4.13.3) | M20A | AB283_1013 |
| ErfK/YbiS/YcfS/YnhG family protein, putative | C82 | AB283_1031, AB283_ 2162 |
| p60 putative peptidase | C40 | AB283_1080 |
| glutamine amidotransferase, class I | C26 | AB283_1128, AB283_2211 |
| Carbamoyl-phosphate synthase small chain (EC 6.3.5.5) | C26 | AB283_1211, AB283_1550 |
| Lipoprotein signal peptidase (EC 3.4.23.36) | A8 | AB283_1213 |
| Signal peptidase I (EC 3.4.21.89) | S26A | AB283_1241, AB283_1612, AB283_2224 |
| Tripeptide aminopeptidase (EC 3.4.11.4) | M20B | AB283_1250, AB283_1855 |
| Sortase A | C60A | AB283_1419 |
| RasP peptidase | M50B | AB283_1437 |
| LexA protease (EC 3.4.21.88) | S24 | AB283_1454 |
| Aminopeptidase YpdF | M24B | AB283_1513 |
| Xaa-Pro dipeptidyl-peptidase (EC 3.4.14.11) | S15 | AB283_1542 |
| Dihydroorotase (EC 3.5.2.3) | M38 | AB283_1551 |
| Membrane-bound protease, CAAX family | M79 | AB283_1655 |
| rhomboid family serine protease | S54 | AB283_1846 |
| Amidophosphoribosyltransferase (EC 2.4.2.14) | C44 | AB283_1899 |
| Phosphoribosylformylglycinamidine synthase | C56 | AB283_1901 |
| stomatin/prohibitin protease | S49 | AB283_1907 |
| Aminopeptidase N | M1 | AB283_1911 |
| D-alanyl-D-alanine carboxypeptidase (EC 3.4.16.4) | S11 | AB283_1944 |
| Cell wall-associated hydrolase | C40 | AB283_2067, AB283_2068, AB283_2069, AB283_2197 |
| oligopeptidase F | M3B | AB283_2084 |
| metallopeptidase | M79 | AB283_2088 |
| Lysyl aminopeptidase (EC 3.4.11.15) | M1 | AB283_2166 |

**Additional file 1:** **Table S3: PTS transporters in *Lactobacillus*** *amylovorus* **JBD401.**

| **Location** | **Specificity** |
| --- | --- |
| AB283_1615, AB283_1827, AB283_203, AB283_2227, AB283_475, AB283_476, AB283_882, AB283_885, AB283_896 | cellobiose |
| AB283_2030, AB283_2031, AB283_717 – AB283_721 | β-glucoside |
| AB283_2044 | Maltose/glucose |
| AB283_2100 | fructose |
| AB283_381 | sucrose |
| AB283_429 – AB283_433 | mannose |
| AB283_446 | mannitol |
| AB283_587 | Maltose |
| AB283_590 | glucose |

**Additional file 1:** **Table S4: ABC transporters in *Lactobacillus*** *amylovorus* **JBD401.**

| **ABC transporter class** | **Location** | **Specificity** |
| --- | --- | --- |
| **FOS** |  |  |
|  | AB283_1822 | msmR |
|  | AB283_380 | bfrA |
|  | AB283_1816 | gtfA |
|  | AB283_1819 | msmG |
|  | AB283_1820 | msmF |
|  | AB283_1818 | msmK |
|  | AB283_1821 | msmE |
| **Ribose** |  |  |
|  | AB283_1598 | Putative deoxyribose-specific ABC transporter |
| **Maltose/maltodextrin** | |  |
|  | AB283_2175 | MalG |
|  | AB283_2176 | MalF |
|  | AB283_2177 | MalE |
|  | AB283_2178 | MalK |
|  | AB283_2184 | MalR |
| **Sugar** |  |  |
|  | AB283_1742 | Sugar transporter |
| **Raffinose** |  |  |
|  | AB283_1816 | gtfAII |
|  | AB283_1817 | melA |
|  | AB283_2103, AB283_2013, AB283_1577, AB283_1618, AB283_1657, AB283_1658, AB283_1745, AB283_2155, AB283_2229, AB283_562, AB283_563 | msmGII |
|  | AB283_1428 | msmRII |
| **Uncharacterized** |  |  |
|  | AB283_2137, AB283_2138, AB283_2168, AB283_2169 | ABC-type multidrug transport system (permease components) |
|  | AB283_867, AB283_ 868 | Multidrug resistance ABC transporter |

**Additional file 1:** **Table S5: Comparison of major genomic features between *L.*** *amylovorus* JBD401 **and *Lactobacillus acidophilus* strains.**

| **Feature** | ***L.*** *amylovorus* **JBD401** | ***L. acidophilus* NCFM** | ***L. acidophilus* 30SC** | ***L. acidophilus* La-14** | ***L. acidophilus* FSI4** |
| --- | --- | --- | --- | --- | --- |
| Genome size (mb) | 1.94 | 1.99 | 2.08 | 1.99 | 1.99 |
| G+C content (%) | 38.1 | 34.7 | 38.1 | 34.7 | 34.7 |
| Genes | 1,979 | 1,927 | 2,084 | 1,948 | 1,948 |
| protein | 1,563 | 1,832 | 1,903 | 1,835 | 1,845 |
| rRNA | 7+1 ncRNA | 13 | 12 | 12 | 12 |
| tRNA | 35 | 61 | 62 | 61 | 61 |
| pseudogene | 372 | 21 | 107 | 40 | 30 |

**Additional file 1:** **Table S6: Comparison of major genomic features between *L.*** *amylovorus* JBD401 **and *Lactobacillus amylovorus* strains.**

| **Feature** | ***L.*** *amylovorus* **JBD401** | ***L. amylovorus* 30SC** | ***L. amylovorus* GRL1118** | ***L. amylovorus* DSM20531** |
| --- | --- | --- | --- | --- |
| Genome size (mb) | 1.94 | 2.08 | 1.89 | 2.17 |
| G+C content (%) | 38.1 | 38.1 | 38.1 | 37.80 |
| Genes | 1,979 | 2,119 | 1,925 | 2,166 |
| protein | 1,563 | 1,904 | 1,762 | 1,891 |
| rRNA | 7 | 12 | 12 | 15 |
| Other RNA | 1 ncRNA | 3 | 3 | 3 |
| tRNA | 35 | 62 | 62 | 64 |
| pseudogene | 372 | 138 | 86 | 193 |

**Additional file 1:** **Table S7: Antibiotic Resistance profile annotation of genome of *Lactobacillus*** *amylovorus* **JBD401.**

| **Best Hit Query Accession** | **Best Hit Accession** | **Best Hit Resistance Type** | **Best Hit Resistance** | **E-Value** | **Percent Identity** | **Cutoff Percent Identity** |
| --- | --- | --- | --- | --- | --- | --- |
| AB283_1752 | ABF68767 | tetw | Tetracycline | 0.0 | 97.65% | 81% |
| AB283_1750 | NP_348076 | tetpb | Tetracycline | 7e-37 | 49.66% | 80% |
| AB283_1806 | ABK86188 | vanra | Teicoplanin, Vancomycin | 3e-11 | 47.56% | 80% |
| AB283_0555 | YP_001334730 | mdtg | Deoxycholate, Fosfomycin | 2e-93 | 45.55% | 80% |
| AB283_2228 | Q83LR7 | macb | Macrolide | 5e-47 | 45.37% | 80% |
| AB283_1425 | AAO43110 | lsa | Lincosamide, Macrolide, Streptogramin_b | 1e-103 | 45.36% | 80% |
| AB283_1993 | AAL27445 | vanre | Vancomycin | 2e-05 | 45.00% | 80% |
| AB283_1617 | Q83LR7 | macb | Macrolide | 3e-46 | 44.91% | 80% |
| AB283_0834 | CAA37477 | otra | Tetracycline | 2e-27 | 43.57% | 80% |
| AB283_2014 | YP_001453760 | macb | Macrolide | 7e-48 | 43.50% | 80% |
| AB283_2136 | ABA71727 | vanrg | Vancomycin | 3e-51 | 42.92% | 80% |
| AB283_0745 | BAE96113 | vanrd | Teicoplanin, Vancomycin | 7e-13 | 42.47% | 80% |
| AB283_1838 | ABS72697 | lmrb | Lincomycin | 1e-104 | 41.72% | 80% |
| AB283_1135 | BAA22228 | lmrb | Lincomycin | 5e-32 | 41.45% | 80% |
| AB283_2220 | ABK86188 | vanra | Teicoplanin, Vancomycin | 8e-26 | 41.22% | 80% |
| AB283_0114 | Q83LR7 | macb | Macrolide | 2e-33 | 40.00% | 80% |

**Additional file 1: Table S8: *In silico* screening of antibiotic resistance for carbamate kinase and ornithine carbamoyltransferase genes.**

|  | **Best Hit Accession** | **Best Hit Resistance Type** | **Best Hit Resistance** | **E-Value** | **Percent Identity** |
| --- | --- | --- | --- | --- | --- |
| **Carbamate kinase** | S60310 | bl2b_tem | Cephalosporin, Penicillin | 1e-21 | 44.85 % |
|  | S60312 | bl2b_tem | Cephalosporin, Penicillin | 0.0 | 43.03 % |
|  | T44117 | mecr1 | Methicillin | 2e-69 | 42.56 % |
| **ornithine carbamoyltransferase** | T44117 | mecr1 | Methicillin | 2e-67 | 43.18 % |
|  | S60312 | bl2b_tem | Cephalosporin, Penicillin | 0.0 | 42.48 % |
|  | S60310 | bl2b_tem | Cephalosporin, Penicillin | 1e-21 | 41.57 % |
